# Isothiocyanates are detected in human synovial fluid following broccoli consumption and can affect the tissues of the knee joint

**DOI:** 10.1038/s41598-017-03629-5

**Published:** 2017-06-13

**Authors:** Rose Davidson, Sarah Gardner, Orla Jupp, Angela Bullough, Sue Butters, Laura Watts, Simon Donell, Maria Traka, Shikha Saha, Richard Mithen, Mandy Peffers, Peter Clegg, Yongping Bao, Aedin Cassidy, Ian Clark

**Affiliations:** 1 School of Biological Sciences, University of East Anglia, Norwich, UK; 2 Institute of Orthopaedics, Norfolk and Norwich University Hospital, Norwich, UK; 3 Institute of Food Research, Norwich, UK; 4 Department of Musculoskeletal Biology, Institute of Ageing and Chronic Disease, University of Liverpool, Liverpool, UK; 5 Norwich Medical School, University of East Anglia, Norwich, UK

## Abstract

Osteoarthritis is a major cause of disability and there is no current pharmaceutical treatment which can prevent the disease or slow its progression. Dietary advice or supplementation is clearly an attractive option since it has low toxicity and ease of implementation on a population level. We have previously demonstrated that sulforaphane, a dietary isothiocyanate derived from its glucosinolate precursor which is found in broccoli, can prevent cartilage destruction in cells, in *in vitro* and *in vivo* models of osteoarthritis. As the next phase of this research, we enrolled 40 patients with knee osteoarthritis undergoing total knee replacement into a proof-of-principle trial. Patients were randomised to either a low or high glucosinolate diet for 14 days prior to surgery. We detected ITCs in the synovial fluid of the high glucosinolate group, but not the low glucosinolate group. This was mirrored by an increase in ITCs and specifically sulforaphane in the plasma. Proteomic analysis of synovial fluid showed significantly distinct profiles between groups with 125 differentially expressed proteins. The functional consequence of this diet will now be tested in a clinical trial.

## Introduction

Osteoarthritis (OA) of the hip or knee is ranked as 11^th^ of 291 conditions that contribute to global disability; and the consequent years lived with disability (YLDs) are estimated to have risen by 61% from 1990–2010^1^. There are no disease-modifying OA drugs (DMOADs) currently available, and pharmacological interventions provide symptomatic relief only, which is frequently insufficient^2, 3^.

The National Institute for Health and Care Excellence (NICE) and the American College of Rheumatology both recommend OA management strategies based upon activity and exercise, and weight loss if overweight or obese. A third core treatment area recommended by NICE focuses on information to support self-management^4, 5^. Within these core treatment areas, dietary and exercise advice have been identified as cost effective approaches to the treatment of OA^6^.

We and others have shown previously that sulforaphane (1-isothiocyanato-4-methylsulfinylbutane, SFN) is a chondroprotective agent *in vitro* and *in vivo* ^7–10^. SFN is derived from a precursor, glucoraphanin, gained from eating brassicas, particularly broccoli. Sulforaphane has broad biological activity and its role in joint health was recently reviewed^11^. SFN can repress the expression of key metalloproteinases implicated in OA^7, 10^, regulate NF-κB and Nrf2 signalling, inhibit production of prostaglandin E2 and nitric oxide in chondrocytes^7, 9, 10^ and prevent cytokine-induced cartilage destruction *in vitro* ^9, 10^. *In vivo*, SFN reduces cartilage destruction in the ‘destabilisation of the medial meniscus’ (DMM) murine model of OA^10^ and inhibits synovial hyperplasia in collagen-induced arthritis (CIA)^8^. Nrf2 knockout mice also showed increased cartilage lesions and oxidative damage in antibody-induced arthritis (AIA)^12^.

The pharmacokinetics of SFN has been studied, with one report measuring the average peak plasma concentration of SFN (2.2 µM) approximately 2 hours following consumption of 100 g of broccoli florets prepared as a soup. This increased to 7.3 µM following consumption of a high glucoraphanin variety of broccoli^13^. There is no evidence that normal dietary intake of broccoli is associated with toxicity, even in the long term. SFN has also been shown to accumulate intracellularly via conjugation to glutathione giving millimolar intracellular concentrations in a cell-type dependent manner^14^. We showed an accumulation of SFN-glutathione (SFN-GSH) in primary chondrocytes of >10-fold and >100-fold in the SW1353 cell line^10^. There are no publications describing the detection of dietary isothiocyanates (ITCs) in the knee joint.

The aim of this study was firstly to determine whether ITCs could be detected in the synovial fluid of OA patients following a glucosinolate (broccoli)-rich diet and secondly to identify changes in the joint tissues.

## Patients and Methods

### Participants

Osteoarthritis patients referred for total knee replacement (TKR) to the Norfolk and Norwich University Hospital (NNUH), Norwich, UK, were screened for recruitment onto the study using the eligibility criteria in Table 1. Eligible patients were male or post-menopausal females scheduled for a TKR willing to comply with the dietary intervention. This study was approved by the National Research Ethics Service Committee East of England, Cambridge South, UK (approval number 2012ORTH06L (104-07-12)). All patients gave informed written consent. All methods were performed in accordance with relevant laboratory guidelines and institutional regulations.

**Table 1:** Eligibility for study screening criteria.

Inclusion criteria	Exclusion criteria
Males and post-menopausal females	Those with known allergies to the dietary compounds/foods/commercially available supplements
OA patient scheduled for total knee replacement	Those unprepared to adhere to dietary restrictions during the trial. i.e. wash out period, consumption of standardised food/commercial supplement
Radiological evidence of some cartilage tissue remaining in the joint to be replaced	Those unwilling to consent to blood sampling or, use of their joint tissues for analysis
Normal biochemical, haematological or urinary assessment as determined by clinical assessor. Full blood count	Participation in another dietary intervention trial running concurrently.
Willingness to comply with dietary requirements	Those taking other food supplements unless they are prepared to abstain during the trial.
	Abnormal biochemical, haematological or urinary assessment indicating abnormal renal or liver function.
	Smokers or those ceasing <3 mths ago
	Pre-menopausal women
	Rheumatoid arthritis, inflammation or sepsis
	Patients taking warfarin medication

Patients scheduled for total knee replacement due to osteoarthritis were screened for eligibility to participate in the study. The inclusion and exclusion criteria are shown. Low glucosinolate diet *n* = 20, high glucosinolate diet *n* = 20.

### Study design

This was a proof-of-principle; randomised controlled trial designed to test whether bioactive molecules derived from broccoli, eaten prior to joint replacement, can be detected in the articular joint and mediate changes.

Patients followed a 7-day washout period, (no consumption of broccoli, cabbage, cauliflower, arugula (rocket), canola/rapeseed, Brussels sprouts, radish, horseradish, kale, turnip root, greens, watercress, pak choi, collard greens, rutabaga (swede) vegetables). The washout diet was either continued (low glucosinolate group) or patients consumed 100 g of a high glucosinolate broccoli^15^ per day for 14 days prior to TKR (high glucosinolate group). Patients were allocated to a high or low glucosinolate group by block randomisation (blocks of five). The trial nurses screened and recruited patients and were blinded to the pre-allocated study numbers. Throughout the study, patients had telephone access to a trial nurse for advice and received a telephone call to monitor compliance.

Blood was sampled at the end of the washout period (baseline) and collected into K_2_EDTA. Patients allocated to the high glucosinolate group were given written instructions, a vegetable steamer and a demonstration for preparation of the broccoli. All patients were given a validated 7-day food diary with guidance for completion. A second blood sample was taken on the day of TKR surgery. Synovial fluid, cartilage and fat tissues were obtained at surgery. Synovial fluid was aspirated into a sterile, pre-chilled container immediately prior to surgery, taking care to avoid blood contamination. Cartilage and fat tissues were removed as routine for TKR and immediately placed into separate containers with pre-chilled, sterile phosphate buffered saline (PBS). All tissue samples were transported at 4 °C in sterile conditions and processed or stored within 1 hour. Synovial fluid was centrifuged at 200 g, 5 minutes at 4 °C and the supernatants stored at −80 °C. Undamaged cartilage was dissected at full thickness from the bone, and both cartilage and fat were snap frozen and stored at −80 °C for RNA extraction, or placed directly into culture for *in vitro* degradation analysis.

Blood plasma and synovial fluid were analysed for ITC levels. Analyses of synovial fluid proteomics, *in vitro* cartilage degradation, joint tissue gene expression profiling and GST genotyping were performed.

### Isothiocyanate detection

Isothiocyanates were detected as described previously^13, 15–17^.

### Isothiocyanate detection: Broccoli

Isothiocyanates were detected as described^15, 16^. No vicinal dithiol contamination of equipment or materials was detected (data not shown). Control samples contained neither broccoli extracts nor 1,2-benzenedithiole (1,2 BDT). High glucosinolate frozen broccoli (100 g) was steamed for 6 mins and blended (0.5 g/ml) in phosphate buffer (33 mM potassium phosphate, 500 µM ascorbic acid, 1 mM EDTA). The broccoli blend was diluted 1:1.5 in distilled water and glucosinolates were hydrolysed using thioglucosidase, 0.05 U/ml (Sigma T4528) at room temperature, 2 hours. The blend was clarified by centrifugation at 3000 g, 5 min and the supernatant was filtered through Whatman no. 41 filter paper, pre-soaked in phosphate buffer. Extraction was repeated three times and pooled. A cyclocondensation reaction of 1,2 BDT and broccoli extracts to 1,3 benzodithiole-2-thione (1,3-BDT) was performed at 65 °C for 2 hours in the dark: the broccoli extract was diluted 1:9 in 100 mM potassium phosphate buffer, pH8.5 and added 1:1 to 8 mM 1,2 BDT dissolved in 100% v/v methanol. The 1,3-benzodithiole-2-thione product was detected at 365 nm.

### Isothiocyanate detection: Plasma

Plasma was deproteinised with a 6% w/v final concentration of polyethylene glycol (PEG) 8000 for 10 mins, at 4 °C, in the dark. Plasma was centrifuged 20 000 g, 5 mins, 4 °C. Supernatant was diluted 1:1 in 100 mM potassium phosphate buffer, pH8.5 with 2x volumes 20 mM 1,2 BDT. Samples were briefly vortexed and then incubated at 65 °C, 2 hours in the dark, followed by centrifugation at 20 000 g for 6 mins, 4 °C. Supernatants were diluted 1:1 in distilled water and purified using solid phase extraction (SPE, C18 Bond Elut, Agilent). The SPE column was activated with 1x vol 100% v/v acetonitrile, 2x vol 25% v/v acetonitile and washed with 2x vols distilled water. Samples were washed x3 using 30% v/v propan-2-ol and eluted with 100% v/v acetonitrile. Reversed phase HPLC was carried out using a Luna® C18 (2) 100 Å, LC Column (5 µm, 150 × 4.60 mm, Phenomenex) at 37 °C using an isocratic mobile phase of 80% v/v methanol, 20% v/v water at a flow rate of 1 ml/min. 1,3 BDT was detected using a SPD-M2OA Prominence HPLC photo diode array detector (Shimadzu) at 365 nm. Sensitivity of the assay in plasma was 6 pmols.

### Sulforaphane detection: Plasma

Plasma samples were analysed for SFN and its conjugates using a method that has been described previously [13] with slight modifications. Briefly plasma samples (100 µl) were prepared by adding 20 µl precooled (4 °C) trichloroacetic acid followed by centrifugation at 11 600 g at 4 °C for 10 min. The injection volume was 5 µl, the HPLC column was a Luna (3 µm particle size, 100 × 2 mm from Phenomenex) using mobile phase flow 0.25 ml/min, the mobile phase were consisted of ammonium acetate buffer (13 mmol/L, pH4; solvent A) and acetonitrile plus 0.1% acetic acid (solvent B) in a linear gradient from 5% B to 35% B over 5 min with 6 min re equilibration time. Agilent 6490 Mass spectrometry analysis was performed with the use of electrospray ionization in positive ion mode with nitrogen gas and nitrogen sheath gas temperatures 160 °C and 400 °C, respectively, gas flow 16 l/min, Nebulizer pressure 30 psi, sheath gas flow 12 l/min and capillary voltage 4000 V.

### Isothiocyanate detection: Synovial fluid

Synovial fluid was diluted in water if necessary and processed as for plasma with the inclusion of at least 10 mins centrifugation following the deproteinisation step. Sensitivity of the assay in synovial fluid was 3.8 pmols.

### Proteomic analysis of synovial fluid

A subset of 18 synovial fluid samples were prepared as described previously^18^. Briefly, samples were hyaluronidase treated, filtered and total protein quantified using Bradford reagent. Synovial fluid samples containing 6 mg total protein were enriched for low abundance proteins using ProteoMiner™ Small Capacity kit (BioRad) according to the manufacturer’s instructions and analysed by SDS-PAGE.

For LC-MS/MS, samples were trypsin digested and loaded on a 2 hour gradient LC-MS/MS analysis using a NanoAcquity™ ultra-performance LC (Waters) and LTQ-Orbitrap-Velos (Thermo-Fisher Scientific)^19^. One sample was excluded for technical reasons (blebbing).

Progenesis™ LC-MS software (Waters, Manchester, UK) was used for label-free quantification^18^. Data were searched using Mascot against the Unihuman Reviewed database. Peptide matches above an identity threshold were adjusted to give a false discovery rate (FDR) of 1% before the protein identifications being re-imported into Progenesis-QI™. Adjusted ANOVA values of p < 0.05 and regulation of >2-fold were regarded as significant. Neopeptides were identified as previously described adjusted to 1% FDR^20^.

### *In vitro* cartilage degradation

Cartilage was dissected into ~5 mm^3^ chips and cultured overnight in quadruplicate in Dulbecco’s modified Eagle’s medium (DMEM; GlutaMAX) supplemented with 1,000 IU/ml penicillin, and 100 µg/ml streptomycin at 37 °C, 5% v/v CO_2_. Culture media were refreshed at day 1 and day 7, with an overall culture of 14 days. Remaining cartilage was papain digested. Culture media and digested cartilage were analysed for glycosaminoglycan and hydroxyproline content as described^21, 22^.

### Gene expression analysis

Cartilage (200 mg) was crushed under liquid nitrogen to a fine powder and total RNA was extracted using TRIzol® reagent (Invitrogen) and RNeasy mini kit (Qiagen), as described^23, 24^.

Approximately 100 mg of fat tissue was homogenised in 600 µl RLT buffer (Qiagen) using the TissueLyser LT (Qiagen) and spherical ball bearings. Total RNA was extracted using the RNeasy mini kit (Qiagen) according to the manufacturer’s instructions.

RNA was quantified using the NanoDrop 2000 spectrophotometer (Thermo Scientific) and the quality assessed using the Experion RNA StdSens analysis kit (Bio-Rad). cDNA was synthesised using 500 ng of RNA and Superscript II reverse transcriptase (Invitrogen) with random hexamers according to the manufacturer’s instructions.

Relative quantification of gene expression was measured using the 7900HT Fast Real Time PCR System (Applied Biosystems) and a custom designed Taqman Low Density Array (TLDA). Genes were selected by analysis of microarray data from SFN treated SW1353 cells, primary human articular chondrocytes and human articular cartilage explants (data not shown). Assay details are included in Supplementary Table S1. Cycling conditions were 50 °C, 20 s, 95 °C, 10 min, 95 °C, 15 s, and 60 °C, 1 min × 40 cycles. Data were analysed using DataAssist Software v3.01 (Life Technologies), normalised to 18 S rRNA and expressed as 2^−ΔCt^.

### Biomarkers

Plasma samples were analysed for Coll 2-1, (a peptide from the triple helix of type II collagen) using ELISA^25^, and hsCRP (high sensitivity C-reactive protein) using a particle-enhanced immunoturbidimetric assay. Analyses were performed by the Bioanalytical Facility (BAF) at the University of East Anglia. Synovial fluid samples were analysed for cytokine protein expression using the Cytometric Bead Array Flex Set (BD Biosciences) and was performed by Flow Cytometry Services, University of East Anglia.

### Diet diary

All patients completed a validated 7-day food diary^26^. Dietary data was analysed using WISP V4.0 (Tinuviel Software).

### Genotyping

DNA was extracted from plasma using QIAamp DNA Blood Mini Kit (Qiagen) according to manufacturer’s instructions. Copy number variation for members of the glutathione S-transferase superfamily, (GSTP1, GSTM1 and GSTT1) was detected using TaqMan® Copy Number Assays (Life Technologies cat: Hs01541162_cn, Hs02575461_cn, Hs00010004) and analysed using CopyCaller® software v2.0 (Life Technologies).

### Data availability

The datasets generated during and/or analysed during the current study are available from the corresponding author on reasonable request.

### Statistical testing

High and low glucosinolate groups were compared using unpaired t-test (two tailed) and when applied, false discovery rate (FDR) was calculated using the Benjamini-Hochberg FDR method. It was noted that within the plasma samples of the low glucosinolate group, all except five samples of the post intervention samples had undetectable levels of SFN. Undetected points were treated as zero for the comparisons of plasma ITCs or SFN, and synovial fluid ITC. Since ITCs were not detected in the low glucosinolate group of the synovial fluid samples, no statistical testing was performed. Differences observed between groups remains the same and conclusions are also identifical using ‘limit of detection’ values instead of zero. All data was analysed using GraphPad Prism version 5.00 for Windows, GraphPad Software, San Diego California USA, unless otherwise stated in the methods. Graphpad Prism calculated descriptive statistics to include confidence intervals and normality testing (D’Agostino & Pearson omnibus normality test). Data with non-Gaussian distribution were transformed prior to further statistical testing. A p value < 0.05 was considered significant.

### Ethics approval

This study protocol was approved by the NRES Committee East of England, Cambridge South, UK.

## Results

### Patients and baseline characteristics

**Figure 1:**
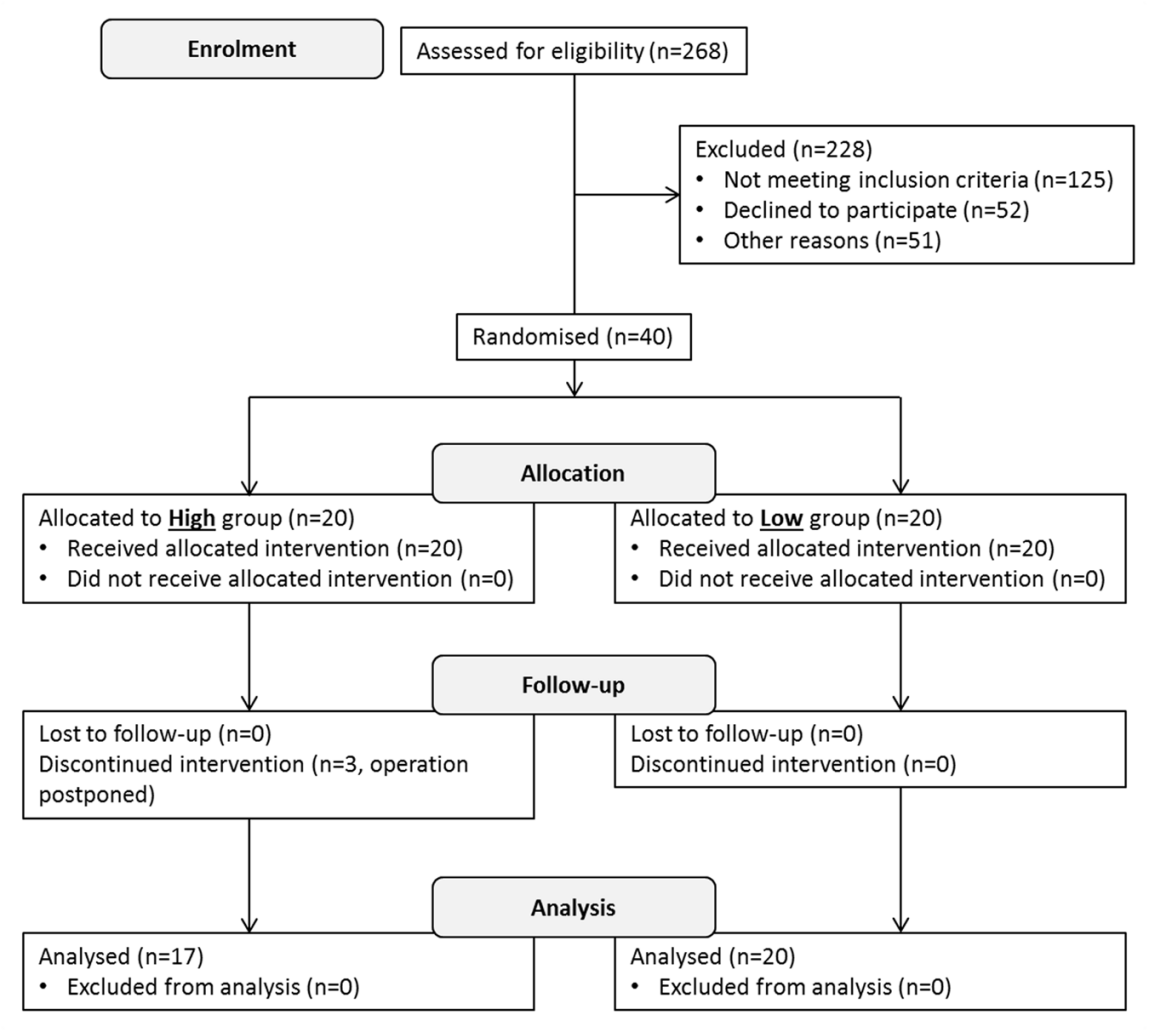
A flow diagram to show the process of enrolment and parallel randomisation for the primary outcome.

A total of 268 patients were screened for eligibility where 143 (53%) were eligible, of which 40 patients (15%) were recruited. A flow diagram to show the process of recruitment and parallel randomisation of the trial is shown in Fig. 1, and more details are given in Supplementary Table S2. Drug use was not recorded. Patient characteristics were males n = 17, (low glucosinolate 7, high glucosinolate 10,), females n = 20 (low glucosinolate 13, high glucosinolate 7). Low glucosinolate group median age 71 (range 50–89), high glucosinolate group median age 73 (range 58–84).

### Isothiocyanate levels in plasma and synovial fluid

Total ITCs measured in the high glucosinolate broccoli were 1.8 µmols g^−1^, so patients consumed 180 µmols per day. Patients in the high glucosinolate group attained greater increases in plasma total ITC concentrations between baseline and post-intervention: high glucosinolate group (mean fold change: 2.20, 95% CI: 1.45 to 2.92) compared to the low glucosinolate group (mean fold change: 0.90, 95% CI: 0.68 to 1.12), p < 0.0001 (Table 2). This confirmed compliance with the intervention. Sulforaphane was also significantly increased in the plasma of the high glucosinolate group (mean change: 0.32, 95% CI: 0.20 to 0.44) compared to low glucosinolate (mean change: 0.02, 95% CI: −0.01 to 0.04), p < 0.0001 (Table 2); since SFN in many post-intervention samples in the low glucosinolate group was undetected and therefore treated as zero, fold change cannot be calculated. Importantly, ITCs were detected in the synovial fluids of the high glucosinolate group (mean 496.6 nM, 95% CI: 419.1 to 574.2) but not in the low glucosinolate group (Table 2). Mean total ITCs and SFN measured in the plasma of the high glucosinolate group (post intervention) were 2.22 µM, 95% CI: 1.19 to 3.24 and 0.32 µM, 95% CI: 0.20 to 0.44 respectively) (Table 2). Data do not segregate on the basis of gender.

**Table 2:** Isothiocyanate levels in synovial fluid and plasma.

Outcome	Low glucosinolate Mean (95%CI)	High glucosinolate Mean (95%CI)	p-value
ITC synovial fluid (nM)	n.d	496.6 (419.10 to 574.20)	—
ITC plasma (fold change)	0.90 (0.68 to 1.12)	2.20 (1.45 to 2.92)	<*0.0001*
SFN plasma (change)	0.02 (−0.01 to 0.04)	0.32 (0.2 to 0.44)	<*0.0001*
ITC plasma (µM) baseline	1.71 (1.10 to 2.33)	1.17 (0.69 to 1.64)	—
ITC plasma (µM) post intervention	1.36 (0.86 to 1.87)	2.22 (1.19 to 3.24)	—
SFN plasma (µM) baseline	n.d	0.004 (−0.004 to 0.012	—
SFN plasma (µM) post intervention	0.02 (−0.005 to 0.04)	0.32 (0.20 to 0.44)	—

Total isothiocyanates (ITCs) measured in synovial fluid and plasma, and the specific metabolite sulforaphane (SFN) measured in plasma from low and high glucosinolate groups. Fold change: post intervention/baseline, Change: post intervention – baseline, low glucosinolate n = 20, high glucosinolate n = 17, n.d – not detected. Mean values with 95% confidence intervals and p-values are given.

### Proteomics

Peptides identified by LC-MS/MS in the synovial fluid samples (low glucosinolate n = 8, high glucosinolate n = 9) were analysed using Progenesis™ QI software (Waters, Manchester, UK). 382 quantifiable proteins were identified, 348 with >1 peptide. One hundred and twenty-five proteins were identified with >1 peptide and differentially expressed between high and low glucosinolate diets (p < 0.05, 1% FDR). An FDR of 5% revealed 29 differentially expressed proteins identified by >1 peptide with a minimum fold change of >2. In the high glucosinolate group 18 proteins had decreased expression whilst 11 proteins were increased compared to the low glucosinolate group. Twelve proteins are commonly found in plasma, 10 of which were decreased in the high glucosinolate group (Table 3). Aside from common plasma proteins, the top four differentially expressed proteins, were neuroblastoma suppressor of tumorigenicity 1 (NBL1), histidine protein methyltransferase 1 homolog (METTL18), calreticulin (CALR) and SWI/SNF complex subunit SMARCC1 (SMARCC1) each p < 0.0001.

**Table 3:** Differentially expressed proteins identified in synovial fluid.

Description	Accession	Peptide count	Unique peptides	q-value	Mean FC (H/L)	Ref
Neuroblastoma suppressor of tumorigenicity 1 (NBL1)	P41271	2	2	1.26E-07	0.33	^34^
Histidine protein methyltransferase 1 homolog (METTL18)	O95568	2	1	2.50E-06	3.29	
Calreticulin (CALR)	P27797	2	2	1.37E-05	0.28	
SWI/SNF complex subunit SMARCC1 (SMARCC1)	Q92922;Q9UL12	2	1	2.17E-05	0.31	
Collagen alpha-1(VI) chain (COL6A1)	P12109	3	2	8.15E-05	0.47	^33, 34^
Adenylyl cyclase-associated protein 1 (CAP1)	Q01518	2	1	1.41E-04	1.0E-06	
Kinesin-like protein KIF26B (KIF26B)	Q2KJY2	3	1	1.08E-03	15.12	
Tubulin alpha-1B chain (TUBA1B)	P68363;A6NHL2;P68366;Q13748;Q71U36	4	4	2.75E-03	2.27	
Adiponectin (ADIPOQ)	Q15848	2	2	3.90E-03	0.19	^34^
Integrin beta-like protein 1 (ITGBL1)	O95965	3	3	5.22E-03	0.39	
Thrombospondin-3 (THBS3)	P49746	3	1	6.51E-03	5.85	^34^
Coronin-1A (CORO1A)	P31146	8	8	6.69E-03	3.47	
Golgin subfamily B member 1 (GOLGB1)	Q14789	3	2	7.33E-03	3.92	
Collagen alpha-1(V) chain (COL5A1)	P20908	3	2	1.03E-02	4.78	^34^
Neutrophil defensin 1 (DEFA1)	P59665	3	3	1.53E-02	3.03	
Ganglioside GM2 activator (GM2A)	P17900	2	2	3.00E-02	3.28	^34^
Tumor necrosis factor-inducible gene 6 protein (TNFAIP6)	P98066	5	5	3.19E-02	0.43	^34^
**Common plasma proteins**						
Apolipoprotein A-II (APOA2)	P02652	8	7	3.82E-09	0.34	^33, 35^
Alpha-1-antitrypsin (SERPINA1)	P01009	17	17	1.52E-07	0.33	^34, 35^
Vitamin K-dependent protein Z (PROZ)	P22891	9	9	1.85E-06	0.32	^34^
Plasma protease C1 inhibitor (SERPING1)	P05155	7	5	7.53E-06	0.41	^34, 35^
Haemopexin (HPX)	P02790	3	3	3.87E-05	0.39	^33, 35^
Apolipoprotein C-I (APOC1)	P02654	2	2	2.79E-04	0.18	^33^
Ig heavy chain V-III region VH26	P01764;P01768	3	1	4.35E-04	0.29	
Ig kappa chain V-II region Cum	P01614;P01617	3	1	4.15E-03	2.14	
Ig kappa chain V-II region RPMI 6410	P06310;P01615;P01616	3	2	6.26E-03	0.34	
Complement factor B (CFB)	P00751	5	5	8.16E-03	0.47	^34, 35^
Fibrinogen gamma chain (FGG)	P02679	28	27	1.14E-02	0.50	^33–36^
Ig mu heavy chain disease protein	P04220	25	2	1.62E-02	5.16	

Differentially expressed proteins in synovial fluid identified by LC-MS/MS. The table shows proteins of interest and common plasma proteins with significantly different levels of expression in low and high glucosinolate groups. Proteins were identified with >1 peptide, >2 fold change and controlled for 5% FDR. The number of peptides identified for each protein and the number of unique peptides for each protein are indicated. Proteins previously identified in the synovial fluid are referenced. Low glucosinolate n = 8, high glucosinolate n = 9. q-values are shown, significance level is q < 0.05. Mean fold change (FC) is given, calculated as peptide abundance in high/low glucosinolate group.

### Neopeptides

Neopeptides identified in synovial fluid unique to high and low glucosinolate groups were identified for aggrecan, biglycan, decorin, fibromodulin, cartilage oligomeric matrix protein and a number of collagens.

There was no correlation between diet and neopeptide abundance, (an indicator of proteolysis), in synovial fluid (see Supplementary Table S3). This holds true whether considering neopeptides unique or common to either intervention.

### *In vitro* cartilage degradation

No significant differences were observed for *in vitro* rates of cartilage degradation (glycosaminoglycan and hydroxyproline loss) between the high and low glucosinolate interventions (Table 4).

**Table 4:** *In vitro* cartilage degradation, biomarkers and cytokine profile.

Outcome	Low glucosinolate Mean (95%CI)	High glucosinolate Mean (95%CI)	p-value
**Cartilage matrix degradation**			
*n*	19	15	
Glycosaminoglycan (% loss)	23.72 (19.58 to 27.87)	25.68 (21.21 to 30.14)	0.50
Hydroxyproline (% loss)	0.71 (0.47 to 0.96)	0.77 (0.50 to 1.03)	0.65
**Biomarkers**			
*n*	20	17	
hsCRP (fold change)	−5.48 (−16.02 to 5.05)	−5.49 (−26.90 to 15.93)	0.33
Col2-1 (fold change)	4.41 (−1.02 to 9.83)	13.21 (−11.85 to 38.27)	0.44
**Cytokine protein expression**			
*n*	19	17	
Interleukin 6 (pg/ml)	84.54 (64.36 to 104.7)	70.42 (53.08 to 87.76)	0.25
Interleukin 8 (pg/ml)	46.62 (30.00 to 63.23)	36.50 (26.94 to 46.07)	0.45
Interleukin 1 (pg/ml)	36.80 (36.59 to 37.00)	36.98 (36.72 to 37.25)	0.25
Tumor necrosis factor (pg/ml)	39.03 (38.70 to 39.37)	39.36 (39.13 to 39.60)	0.10
Chemokine (C-C Motif) ligand 5 (pg/ml)	54.04 (43.39 to 64.69)	66.80 (31.05 to 102.6)	0.68

*In vitro* degradation of cartilage aggrecan and collagen were measured by glycosaminoglycan and hydroxyproline release into culture media. Remaining cartilage was digested and percentage degradation calculated. High sensitivity C reactive protein (hsCRP) and collagen type II (col 2-1) biomarkers for inflammation and collagen degradation were measured in plasma (mean fold change given). The protein expression levels of cytokines were profiled in synovial fluid. Mean values and 95% confidence intervals are shown. P-values are given and the significance level was p < 0.05.

### Gene Expression

Gene expression was measured by TLDA in cartilage (low glucosinolate n = 20, high glucosinolate n = 17) and fat (low glucosinolate n = 13, high glucosinolate n = 10) tissues. Adjustment for multiple testing by controlling the false discovery rate (5%) generated no significant differences between groups for either tissue (Supplementary Table S4).

However, unadjusted data showed that in cartilage *HERC5* (HECT and RLD Domain Containing E3 Ubiquitin Protein Ligase 5) (p = 0.009) and *LUM* (lumican) (p = 0.05) were decreased in the high glucosinolate group. *IL-1β* (interleukin 1β) (p = 0.01) and *IGFBP4* (Insulin-Like Growth Factor Binding Protein 4) (p = 0.03) were increased in the high glucosinolate group. Taking large fold changes into account *CCL7* (Chemokine (C-C Motif) Ligand 7) (p = 0.06) and *SFRP1* (Secreted Frizzled-Related Protein 1) (p = 0.06) showed tendencies to decreased and increased expression respectively in the high glucosinolate group. In fat tissue *CXCL10* (Chemokine (C-X-C Motif) Ligand 10) (p = 0.03) was decreased in the high glucosinolate group whilst *IRX3* (Iroquois Homeobox 3) (p = 0.06) was increased.

### Plasma Biomarkers – hsCRP and Coll 2-1

The 14-day intervention did not alter levels of either hsCRP (with many samples undetected) or Coll 2-1 in plasma, or protein cytokine expression of IL-6, IL-8, IL-1, TNF, CCL5, in synovial fluid (Table 4).

### Diet Diaries

Nutrient intakes were largely similar between groups however, a significant increased intake of vitamin C (p = 9.96 × 10^−06^) was observed following adjustment for multiple testing using a 1% FDR (Supplementary Table S5).

### Genotyping

The overall frequency of GSTT1 copy number was measured at 3 (8.1%) 0 copies, 27 (73%) 2 copies, and 7 (18.9%) 4 copies. The overall frequency of GSTM1 copy number was measured at: 17 (45.9%) 0 copies, 18 (48.6%) 2 copies, and 2 (5.4%) 4 copies. The overall frequency of GSTP1 copy number was measured at: 18 (48.6%) 2 copies, 7 (18.9%) 3 copies, 10 (27%) 4 copies and 1 (2.7%) 5 copies. Predicted copy numbers were similar between the high and low glucosinolate groups and are detailed in Supplementary Table S6. No correlation was observed between genotype and any outcome measure.

### Adverse events

There was one serious adverse event of a fractured humerus considered unrelated to the intervention. In terms of minor events, one participant reported bloating (high glucosinolate group), although the participant continued with the intervention.

## Discussion

Our results provide the first evidence that following broccoli intake, the bioactive constituent ITCs reach the synovial fluid at concentrations with biological impact on the articular joint tissues, and alter the synovial fluid protein profile. This study clearly demonstrates that a dietary bioactive with chondroprotective properties can penetrate the knee in osteoarthritis. These data support our previous mouse model work, where we showed that a SFN-rich diet can provide chondroprotection in a mouse model of OA, though SFN and its metabolites were not measured directly in the joint.

Mean total ITC levels were 4.47-fold lower in the synovial fluid than the plasma. The concentration of small molecules in synovial fluid often reflects that of plasma since permeability into synovial fluid occurs through free diffusion from the endothelium of the synovial microvasculature and surrounding interstitium. The synovial lining has no basement membrane and therefore small molecule diffusion is limited only by the volume of intercellular space^27^, however, many non-steroidal anti-inflammatory drugs also show lower concentrations in synovial fluid than plasma^28, 29^. This reflects the kinetics of elimination of the drug, repeat administration and also protein binding. In this study we did not standardize the timing of broccoli intake compared to sampling of blood or synovial fluid. It is also possible that the diet modifies synovial fluid volume which was not monitored in this study, though this is unlikely to be significant. Plasma levels for ITCs and SFN were higher than expected given the limited available published data. In the current study the timing of the broccoli meal during the day was not controlled. This and possible differences in the rate of metabolism by each patient’s unique microbiome^30^ may explain the greater variation observed for plasma ITC levels in the high glucosinolate group. Twelve of the 29 synovial fluid proteins with significant difference between groups are commonly found in plasma, and 10 of these were decreased in the high glucosinolate group. In the inflamed joint, changes in the permeability of the synovium have been described, whereby increases in protein and solute concentration within the joint cavity have been observed^31, 32^. Resolution of aberrant synovial permeability by the high glucosinolate diet may offer one possible explanation for a reduction in the number of plasma proteins observed in this group, though this remains to be confirmed. Furthermore, a number of the proteins highlighted in our study (table 3) have been reported in human OA synovial fluid previously^33–36^.

Common plasma proteins aside, there were a number of interesting proteins decreased in abundance with the high glucosinolate diet including NBL1, CALR, SMARCC1 and type VI collagen. NBL1 is an antagonist of bone morphogenetic proteins (BMPs), key in cartilage homeostasis. Increased gene expression of NBL1 in cartilage is reported in mouse and rat models of OA and in human OA cartilage^37–40^. Calreticulin has functions outside of calcium regulation^41^. Calreticulin is increased in the plasma and synovial fluid of RA patients and this correlated with swollen joint count and disease activity score^42^. SMARCC1 is a regulator of gene transcription through chromatin remodelling, and is found in exosomes in cancer with no reported function in the joint^43–45^. Type VI collagen is a structural component of the perichondral matrix within articular cartilage. It has been reported in synovial fluid, with no significant difference between OA and control^33^. Methyltransferase-like 18 (METTL18) was significantly increased in abundance in the high glucosinolate group. It is a putative methyltransferase, and interacts with molecular chaperones^46^. Individual neopeptides were not reproducibly identified across all samples and this technology requires further development to be conclusive.

Gene expression in either cartilage or fat was not significantly different between groups when data were adjusted for multiple testing. Genes shown to be significant in unadjusted data are therefore of uncertain importance.

The collagen degradation biomarker Coll 2-1 has previously been shown to decrease after supplementation with a form of curcumin^47^, however only at the 85 day time point and not at the earlier 14 day time point.

Validated diet diaries showed that patients consumed similar levels of energy, fat, protein and fibre during the intervention. The high glucosinolate group consumed significantly higher levels of vitamin C, likely due to the high vitamin C content of broccoli. We therefore cannot rule out the contribution of vitamin C to the effects observed in the study. Future studies involving broccoli intake should include a control for vitamin C levels.

The interaction of GST genotype and ITC metabolism measured as rate of urinary secretion has been reported previously with differing associations^13, 48–50^. It has been suggested that specific GST null genotypes may confer a protective effect through a lowered rate of metabolism that extends tissue exposure time. Our cohort showed no associations of genotype with study outcomes though numbers in each subgroup were small (data not shown).

### Limitations

Our study was a proof-of-principle study and limitations of the trial design were the short intervention time and unspecified timing of broccoli intake. These limitations and/or the use of end-stage OA patients whose tissue gene expression maybe beyond modification by dietary intervention, may have contributed to the small changes in gene expression observed. Similarly, cartilage degradation rates *in vitro* were likely affected by rapid metabolism of dietary SFN in these cultures. The link between ITCs in the joint and changes in the synovial fluid may be indirect and this requires further investigation.

## Conclusion

This is the first human study to show that increased broccoli intake results in ITC uptake into the joint, with concomitant changes in the joint. Coupled with our earlier data showing efficacy of SFN in both *in vitro* and *in vivo* laboratory models of OA, this supports the need for an appropriately designed clinical trial to determine the impact of dietary SFN in OA.

## Electronic supplementary material

Supplementary information
