# Supplementary material for: Isothiocyanates are detected in human synovial fluid following broccoli consumption and can affect the tissues of the knee joint
Source: Sci Rep. 2017 Jun 13;7:3398. doi: 10.1038/s41598-017-03629-5 (PMC5469854; doi:10.1038/s41598-017-03629-5)
Supplement: Supplementary file 1 — Supplementary information [file 41598_2017_3629_MOESM1_ESM.pdf]

SUPPLEMENTARY INFORMATION

Isothiocyanates are detected in human synovial fluid following broccoli consumption and can affect the tissues of the knee joint.

Dr. Rose Davidson Ph.D[1], Miss Sarah Gardner BSc[1], Dr. Orla Jupp Ph.D[1], Mrs. Angela Bullough BSc[2], Mrs. Sue Butters BSc[2], Mrs. Laura Watts MSc[2], Prof. Simon Donell MD[2], Dr. Maria Traka Ph.D[3], Dr. Shikha Saha Ph.D[3], Prof. Richard Mithen Ph.D[3], Dr. Mandy Peffers Ph.D[4], Prof. Peter Clegg Ph.D[4], Dr. Yongping Bao Ph.D[5], Prof. Aedin Cassidy Ph.D[5], Prof. Ian Clark Ph.D[1]*.

[1]School of Biological Sciences, University of East Anglia, Norwich, UK. [2]Institute of Orthopaedics, Norfolk and Norwich University Hospital, Norwich, UK. [3]Institute of Food Research, Norwich, UK. [4]Department of Musculoskeletal Biology, Institute of Ageing and Chronic Disease, University of Liverpool, Liverpool, UK. [5]Norwich Medical School, University of East Anglia, Norwich, UK

Corresponding author: *Ian M. Clark

Supplementary file S1		Davidson et al 2017
Detector	Reporter	Quencher
18S-Hs99999901_s1	FAM	Non Fluorescent
ADAMTS1-Hs00199608_m1	FAM	Non Fluorescent
BMP6-Hs01099594_m1	FAM	Non Fluorescent
BST2-Hs01561315_m1	FAM	Non Fluorescent
CCL5-Hs00982282_m1	FAM	Non Fluorescent
CCL8-Hs04187715_m1	FAM	Non Fluorescent
COL1A2-Hs01028969_m1	FAM	Non Fluorescent
COL4A1-Hs00266237_m1	FAM	Non Fluorescent
CXCL9-Hs00171065_m1	FAM	Non Fluorescent
CCL20-Hs01011368_m1	FAM	Non Fluorescent
CCL7-Hs00171147_m1	FAM	Non Fluorescent
CD274-Hs01125301_m1	FAM	Non Fluorescent
CPXM1-Hs00219709_m1	FAM	Non Fluorescent
GBP3-Hs00544385_m1	FAM	Non Fluorescent
GCLM-Hs00157694_m1	FAM	Non Fluorescent
IFI27-Hs01086370_m1	FAM	Non Fluorescent
IFI6-Hs00242571_m1	FAM	Non Fluorescent
IFI6-Hs01564159_m1	FAM	Non Fluorescent
IGFBP4-Hs01057900_m1	FAM	Non Fluorescent
IL1A-Hs00174092_m1	FAM	Non Fluorescent
IL1B-Hs01555410_m1	FAM	Non Fluorescent
LCN2-Hs01008571_m1	FAM	Non Fluorescent
MARCKS-Hs00158993_m1	FAM	Non Fluorescent
MMP3-Hs00968305_m1	FAM	Non Fluorescent
NQO1-Hs01045994_m1	FAM	Non Fluorescent
EMILIN1-Hs00915216_mH	FAM	Non Fluorescent
FAIM3-Hs01056796_m1	FAM	Non Fluorescent
FCAMR-Hs01049677_m1	FAM	Non Fluorescent
GBP2-Hs00894837_m1	FAM	Non Fluorescent
HERC5-Hs01061812_m1	FAM	Non Fluorescent
HMOX1-Hs01110250_m1	FAM	Non Fluorescent
ICAM1-Hs00164932_m1	FAM	Non Fluorescent
IFI44L-Hs00915292_m1	FAM	Non Fluorescent
IFI44-Hs00951349_m1	FAM	Non Fluorescent
IFIT2-Hs00533665_m1	FAM	Non Fluorescent
IL15RA-Hs00542604_m1	FAM	Non Fluorescent
IL36G-Hs00219742_m1	FAM	Non Fluorescent
IL6-Hs00985639_m1	FAM	Non Fluorescent
IL8-Hs00174103_m1	FAM	Non Fluorescent
IRF1-Hs00971960_m1	FAM	Non Fluorescent
LRP8-Hs00902382_m1	FAM	Non Fluorescent
ME1-Hs00159110_m1	FAM	Non Fluorescent
MMP12-Hs00899662_m1	FAM	Non Fluorescent
MMP13-Hs00233992_m1	FAM	Non Fluorescent

OASL-Hs00984387_m1	FAM	Non Fluorescent
PARP14-Hs00981511_m1	FAM	Non Fluorescent
PENK-Hs00175049_m1	FAM	Non Fluorescent
PIM2-Hs00179139_m1	FAM	Non Fluorescent
PIR-Hs01125822_m1	FAM	Non Fluorescent
POPDC3-Hs01592415_m1	FAM	Non Fluorescent
PTX3-Hs01073991_m1	FAM	Non Fluorescent
SELE-Hs00950401_m1	FAM	Non Fluorescent
SFRP1-Hs00610060_m1	FAM	Non Fluorescent
SLC15A3-Hs00275455_m1	FAM	Non Fluorescent
SLC40A1-Hs00205888_m1	FAM	Non Fluorescent
TNFSF10-Hs00921974_m1	FAM	Non Fluorescent
TRAF1-Hs01090169_m1	FAM	Non Fluorescent
TXNRD1-Hs00917067_m1	FAM	Non Fluorescent
UBD-Hs00197374_m1	FAM	Non Fluorescent
UBE2L6-Hs01125548_m1	FAM	Non Fluorescent
S100A4-Hs00243202_m1	FAM	Non Fluorescent
TSG6-Hs01113602_m1	FAM	Non Fluorescent
CXCL10-AJN1FLP	FAM	Non Fluorescent
IRX3-AJKAK3C	FAM	Non Fluorescent
TNFRSF4-AJ70MDM	FAM	Non Fluorescent
TGFBI-Hs00932745_m1	FAM	Non Fluorescent
COL5A1-Hs00609133_m1	FAM	Non Fluorescent
THBS3-Hs00938498_m1	FAM	Non Fluorescent
CTSD-Hs00157205_m1	FAM	Non Fluorescent
FN1-Hs00415006_m1	FAM	Non Fluorescent
PRG4-Hs00981633_m1	FAM	Non Fluorescent
COMP-Hs00164359_m1	FAM	Non Fluorescent
HSPG2-Hs00194179_m1	FAM	Non Fluorescent
HABP2-Hs00188053_m1	FAM	Non Fluorescent
A2M-Hs00929971_m1	FAM	Non Fluorescent
LUM-Hs00929860_m1	FAM	Non Fluorescent
THBS4-Hs00170261_m1	FAM	Non Fluorescent
GPX3-Hs01078668_m1	FAM	Non Fluorescent
SAA4-Hs00197854_m1	FAM	Non Fluorescent
HAPLN1-Hs01091999_m1	FAM	Non Fluorescent
SOD3-Hs00162090_m1	FAM	Non Fluorescent
FMOD-Hs00157619_m1	FAM	Non Fluorescent
TNFAIP6-Hs01113602_m1	FAM	Non Fluorescent
COL6A1-Hs01095585_m1	FAM	Non Fluorescent
OGN-Hs00247901_m1	FAM	Non Fluorescent
ITIH3-Hs00158314_m1	FAM	Non Fluorescent
HTRA1-Hs01016151_m1	FAM	Non Fluorescent
SERPIND1-Hs00164821_m1	FAM	Non Fluorescent
ITGBL1-Hs01557019_m1	FAM	Non Fluorescent
ADIPOQ-Hs00977214_m1	FAM	Non Fluorescent
MMP2-Hs01548727_m1	FAM	Non Fluorescent
RPL13A-Hs01578912_m1	FAM	Non Fluorescent
YWHAZ-Hs00237047_m1	FAM	Non Fluorescent
GDF11-Hs00195156_m1	FAM	Non Fluorescent
TNFSF11-Hs00243522_m1	FAM	Non Fluorescent

Supplementary S1. Assays for gene expression (shown) were supplied by Applied Biosystems and used to obtain custom Taqman Low Density Arrays. FAM, 6-carboxyfluorescein.

**Eligible**		**Not Eligible**	
Reason for Exclusion	Number	Reason for Exclusion	Number
Unable to contact	12	Smoker	14
Unsuitable TKR date	34	Rheumatoid Arthritis	10
Decline-No reason given	10	Inflammation/Infection	20
Decline-Not interested	34	Hormone Therapy/Steroids	12
Decline-Time factors	8	No freezer capacity	6
Recruited	40	Doesn't like broccoli	28
Not contacted-end of study	5	Unable to adhere to diet	12
		Unprepared to adhere to diet	8
		Already recruited	6
		Not for TKR	6
		Unwilling to comply with sampling	1
**Subtotal**	123		143
**Total**	266		

Supplementary S2. Study Recruitment details. In total 266 patients scheduled for total knee replacement were screened using the eligibility criteria (table 1). Eligible and non-eligible patients (numbers shown) were excluded from the study for the reasons given in the above table.

Accession	Protein	Previous	Peptide sequence	Next	Abundance		FOLD CHANGE	P VALUE
					LOW	HIGH		
P20908	Collagen alpha-1(V) chain OS=Homo sapiens GN=COL5A1	K	GPQGPAGRDGLQGPVGLPGPAG	P	0.382	0.051	0.132	5.59E-09
P02452	Collagen alpha-1(I) chain OS=Homo sapiens GN=COL1A1	G	EPGPTGLPGPPGER	G	0.013	0.002	0.170	3.35E-08
P20908	Collagen alpha-1(V) chain OS=Homo sapiens GN=COL5A1	K	GEPGDVGPQGPRGVQGPPG	P	20.002	4.165	0.208	5.05E-08
P20908	Collagen alpha-1(V) chain OS=Homo sapiens GN=COL5A1	L	KGNEGPPGPPGPAGSPGER	G	20.002	4.165	0.208	5.05E-08
P02452	Collagen alpha-1(I) chain OS=Homo sapiens GN=COL1A1	N	AGPPGPPGPAGK	E	0.102	0.022	0.215	7.26E-08
P02452	Collagen alpha-1(I) chain OS=Homo sapiens GN=COL1A1	G	APGPSGARGER	G	0.006	0.002	0.249	1.59E-07
P16112	Aggrecan core protein OS=Homo sapiens GN=ACAN	R	LEQFTFQEAL	E	0.378	0.081	0.214	1.22E-06
P02452	Collagen alpha-1(I) chain OS=Homo sapiens GN=COL1A1	P	GDRGEPGPPGPAGFAGPPGADGQPGAK	G	1.337	0.170	0.127	1.76E-06
P20908	Collagen alpha-1(V) chain OS=Homo sapiens GN=COL5A1	G	ITGKPGPK	G	0.106	0.019	0.178	2.44E-06
P02452	Collagen alpha-1(I) chain OS=Homo sapiens GN=COL1A1	R	GETGPAGPTGP	V	0.136	0.024	0.176	3.37E-06
P02452	Collagen alpha-1(I) chain OS=Homo sapiens GN=COL1A1	P	GAPGPQGFQGPPGEPGEPGASGPMGPRGPPGPPGK	N	1.002	0.097	0.096	7.09E-06
P02461	Collagen alpha-1(III) chain OS=Homo sapiens GN=COL3A1	R	NGDPGIPGQPGSPGSP	G	0.003	0.001	0.230	7.70E-06
P02461	Collagen alpha-1(III) chain OS=Homo sapiens GN=COL3A1	T	GPIGPPGPAGQPGDK	G	0.612	0.138	0.226	7.94E-06
P02452	Collagen alpha-1(I) chain OS=Homo sapiens GN=COL1A1	D	AGAPGAPGSQGAPGLQGMPGER	G	0.018	0.005	0.266	1.31E-05
P20908	Collagen alpha-1(V) chain OS=Homo sapiens GN=COL5A1	R	AAYDYCEHYSPDCDTAVPDTPQSQDPNP	D	0.369	0.149	0.402	1.31E-05
P02452	Collagen alpha-1(I) chain OS=Homo sapiens GN=COL1A1	R	GVQGPPGPAGP	R	0.095	0.031	0.322	1.41E-05
P02452	Collagen alpha-1(I) chain OS=Homo sapiens GN=COL1A1	R	TGDAGPVGPPG	P	0.300	0.094	0.312	2.08E-05
P02461	Collagen alpha-1(III) chain OS=Homo sapiens GN=COL3A1	N	DGARGSDGQPGPPGPPGTAGFPGSPGAK	G	1.124	0.056	0.050	2.21E-05
P02452	Collagen alpha-1(I) chain OS=Homo sapiens GN=COL1A1	G	PPGPPGPAGEK	G	0.145	0.054	0.372	3.37E-05
P20908	Collagen alpha-1(V) chain OS=Homo sapiens GN=COL5A1	K	TGPIGPQGAPGKPGPDG	L	2.213	0.843	0.381	6.02E-05
P02461	Collagen alpha-1(III) chain OS=Homo sapiens GN=COL3A1	R	GSPGPQGVKGESGKP	G	0.002	0.001	0.510	9.16E-05
P02452	Collagen alpha-1(I) chain OS=Homo sapiens GN=COL1A1	K	NCPGAEVPEGECCPVCP	D	0.117	0.023	0.198	9.25E-05
P20908	Collagen alpha-1(V) chain OS=Homo sapiens GN=COL5A1	K	GPLGKPGLPGMPGADGPPGHPGKEGPP	G	2.564	0.030	0.012	1.09E-04
P02461	Collagen alpha-1(III) chain OS=Homo sapiens GN=COL3A1	R	GPPGPQGLPGL	A	4.018	1.104	0.275	1.13E-04
P02461	Collagen alpha-1(III) chain OS=Homo sapiens GN=COL3A1	D	GTSGHPGPIGPPGPR	G	0.788	0.154	0.195	1.36E-04
P20908	Collagen alpha-1(V) chain OS=Homo sapiens GN=COL5A1	P	GKEGPPGEK	G	5.071	0.330	0.065	1.36E-04
P20908	Collagen alpha-1(V) chain OS=Homo sapiens GN=COL5A1	K	GDKGEQGPPGPTGPQGPIGQPGPSGADGEPGP	R	4.256	0.690	0.162	1.45E-04
P02452	Collagen alpha-1(I) chain OS=Homo sapiens GN=COL1A1	G	VRGEPGPPGPAGAAGPAGNPGADGQPGAK	G	3.286	0.641	0.195	1.52E-04
P02452	Collagen alpha-1(I) chain OS=Homo sapiens GN=COL1A1	K	GDRGETGPAGPPGAPGAPGAPGPV	G	0.464	0.235	0.506	1.53E-04
P02452	Collagen alpha-1(I) chain OS=Homo sapiens GN=COL1A1	G	APGIAGAPGFPGARGPSGPQGPGGPPGPK	G	0.430	0.047	0.109	1.56E-04
P02452	Collagen alpha-1(I) chain OS=Homo sapiens GN=COL1A1	G	PRGPAGPPGR	D	0.030	0.011	0.356	1.69E-04
P20908	Collagen alpha-1(V) chain OS=Homo sapiens GN=COL5A1	K	DLQLCHPDFPDGEYWVDP	N	10.558	3.056	0.289	2.01E-04
P02461	Collagen alpha-1(III) chain OS=Homo sapiens GN=COL3A1	G	PPGPSGPRGQPGVMGFPGPK	G	0.110	0.022	0.197	2.04E-04
P02452	Collagen alpha-1(I) chain OS=Homo sapiens GN=COL1A1	R	GVPGPPGAVGPAG	K	0.281	0.122	0.432	2.06E-04
P02461	Collagen alpha-1(III) chain OS=Homo sapiens GN=COL3A1	R	GENGSPGAPGAPGHPGPPGPVGPAG	K	12.392	4.575	0.369	2.07E-04
P02452	Collagen alpha-1(I) chain OS=Homo sapiens GN=COL1A1	G	PSGPAGPTGAR	G	0.085	0.031	0.366	2.47E-04
P20908	Collagen alpha-1(V) chain OS=Homo sapiens GN=COL5A1	S	PGPDGPPGPMGPPGLPGLKGDSGPK	G	2.857	0.085	0.030	2.75E-04
P02461	Collagen alpha-1(III) chain OS=Homo sapiens GN=COL3A1	P	PGSNGNPGPPGPSGSPGK	D	0.005	0.002	0.326	3.09E-04
P02461	Collagen alpha-1(III) chain OS=Homo sapiens GN=COL3A1	R	GLPGPPGSNGNPGPPGPSGSPGKDGPP	G	0.016	0.002	0.101	3.32E-04
P20908	Collagen alpha-1(V) chain OS=Homo sapiens GN=COL5A1	Q	GLPGLAGKEGTK	G	0.003	0.001	0.213	3.63E-04
P02452	Collagen alpha-1(I) chain OS=Homo sapiens GN=COL1A1	D	GSPGKDGVR	G	0.056	0.016	0.285	3.76E-04
P20908	Collagen alpha-1(V) chain OS=Homo sapiens GN=COL5A1	I	GPPGEKGPLGKPGLPGMPGADGPPGHPGK	E	89.489	8.247	0.092	4.79E-04
P02452	Collagen alpha-1(I) chain OS=Homo sapiens GN=COL1A1	R	GFSGLQGPPGPPGSPGEQGPSGASGPAGPRGPPGS	A	0.486	0.030	0.061	5.44E-04
P02452	Collagen alpha-1(I) chain OS=Homo sapiens GN=COL1A1	R	TGDAGPVGPPGPPGP	P	0.036	0.008	0.229	5.55E-04
P02461	Collagen alpha-1(III) chain OS=Homo sapiens GN=COL3A1	K	SGDRGESGPAGPAGAPGPA	G	5.699	1.794	0.315	5.68E-04
P02452	Collagen alpha-1(I) chain OS=Homo sapiens GN=COL1A1	R	GLTGPIGPPGPA	G	0.001	0.000	0.531	6.32E-04
P02461	Collagen alpha-1(III) chain OS=Homo sapiens GN=COL3A1	R	GEPGPQGHAGAQGPPGP	P	0.238	0.044	0.185	7.33E-04
P02452	Collagen alpha-1(I) chain OS=Homo sapiens GN=COL1A1	K	AGERGVPG	P	0.023	0.012	0.516	7.82E-04
P02452	Collagen alpha-1(I) chain OS=Homo sapiens GN=COL1A1	P	GPAGFAGPPGADGQPGAKGEPGDAGAK	G	15.407	3.668	0.238	7.90E-04
P02461	Collagen alpha-1(III) chain OS=Homo sapiens GN=COL3A1	K	GAAGPPGPPGAAGTPGLQGMPGERGGLGS	P	4.346	0.088	0.020	9.17E-04
P02461	Collagen alpha-1(III) chain OS=Homo sapiens GN=COL3A1	K	GEMGPAGI	P	0.259	0.077	0.299	9.49E-04
P02461	Collagen alpha-1(III) chain OS=Homo sapiens GN=COL3A1	S	PGGKGEMGPAGIPGAPGLMGAR	G	13.034	3.551	0.272	1.01E-03
P20908	Collagen alpha-1(V) chain OS=Homo sapiens GN=COL5A1	P	GKTGPIGPQGAPGKPGPDGLR	G	0.527	0.167	0.317	1.02E-03
P02452	Collagen alpha-1(I) chain OS=Homo sapiens GN=COL1A1	P	GPPGPPGPPGPPSAGFDFSFLPQPPQEK	A	0.084	0.012	0.146	1.17E-03
P20908	Collagen alpha-1(V) chain OS=Homo sapiens GN=COL5A1	I	TGPSGPIGPPGPPGLPGPPGPKGAK	G	2.553	0.184	0.072	1.17E-03
P02452	Collagen alpha-1(I) chain OS=Homo sapiens GN=COL1A1	K	GEPGPVGVQGP	P	0.043	0.025	0.590	1.21E-03
P20908	Collagen alpha-1(V) chain OS=Homo sapiens GN=COL5A1	K	GSPGPVGFPGDPGPPGEPGPAGQDGPP	G	0.012	0.005	0.389	1.44E-03
P02461	Collagen alpha-1(III) chain OS=Homo sapiens GN=COL3A1	P	GSNGAPGQR	G	0.015	0.006	0.412	1.52E-03
P02461	Collagen alpha-1(III) chain OS=Homo sapiens GN=COL3A1	P	PGIKGPAGIPGFPGMK	G	78.065	18.196	0.233	1.58E-03
P20908	Collagen alpha-1(V) chain OS=Homo sapiens GN=COL5A1	P	GEPGPRGLLGPK	G	4.702	3.061	0.651	1.87E-03
P20908	Collagen alpha-1(V) chain OS=Homo sapiens GN=COL5A1	R	GPSGAPGADGPQGPPGG	I	7.462	2.015	0.270	1.99E-03
P02452	Collagen alpha-1(I) chain OS=Homo sapiens GN=COL1A1	G	VPGPPGAVGPAGK	D	0.163	0.058	0.354	2.01E-03
P02452	Collagen alpha-1(I) chain OS=Homo sapiens GN=COL1A1	G	PSGNAGPPGPPGPAGK	E	0.027	0.003	0.122	2.08E-03
P02461	Collagen alpha-1(III) chain OS=Homo sapiens GN=COL3A1	P	IGPPGPAGQPGDK	G	0.632	0.214	0.338	2.27E-03
P20908	Collagen alpha-1(V) chain OS=Homo sapiens GN=COL5A1	P	TGKPGPK	G	2.610	0.891	0.341	2.38E-03
P20908	Collagen alpha-1(V) chain OS=Homo sapiens GN=COL5A1	Q	DAATGSYDKALR	F	1.141	0.724	0.635	2.44E-03
P02452	Collagen alpha-1(I) chain OS=Homo sapiens GN=COL1A1	K	GPAGERGSPGPA	G	0.005	0.002	0.390	2.50E-03
P02452	Collagen alpha-1(I) chain OS=Homo sapiens GN=COL1A1	P	PGPMGPPGLAGPPGESGR	E	0.078	0.021	0.271	3.00E-03
P20908	Collagen alpha-1(V) chain OS=Homo sapiens GN=COL5A1	K	GNSGGDGPAGPPGERGPNGPQGPT	G	1.904	0.414	0.218	3.20E-03
P02461	Collagen alpha-1(III) chain OS=Homo sapiens GN=COL3A1	G	PAGPRGPVGPSGPPGK	D	0.698	0.357	0.511	3.20E-03
P20908	Collagen alpha-1(V) chain OS=Homo sapiens GN=COL5A1	P	GVTGMDGQPGPK	G	0.408	0.153	0.375	3.29E-03
P20908	Collagen alpha-1(V) chain OS=Homo sapiens GN=COL5A1	I	GLQGLPGPPGEK	G	0.036	0.015	0.421	3.31E-03
P02461	Collagen alpha-1(III) chain OS=Homo sapiens GN=COL3A1	G	GPGADGVPGK	D	0.075	0.031	0.416	3.38E-03
P02452	Collagen alpha-1(I) chain OS=Homo sapiens GN=COL1A1	G	APGDRGEPGPPGPAGFAGPPGADGQPGAK	G	2.434	0.712	0.293	3.42E-03
P02452	Collagen alpha-1(I) chain OS=Homo sapiens GN=COL1A1	K	GPRGETGPAGRPGEVGPPG	P	0.005	0.002	0.449	3.73E-03
P20908	Collagen alpha-1(V) chain OS=Homo sapiens GN=COL5A1	G	PPGPAGEKGAPGEK	G	0.006	0.002	0.324	3.79E-03
P20908	Collagen alpha-1(V) chain OS=Homo sapiens GN=COL5A1	G	FPGPPGPVGLQGLPGPPGEK	G	2121.023	181.427	0.086	4.00E-03
P02461	Collagen alpha-1(III) chain OS=Homo sapiens GN=COL3A1	R	GLPGPPGIKGP	A	0.235	0.106	0.451	4.09E-03
Q06828	Fibromodulin OS=Homo sapiens GN=FMOD	R	SAMPADAPL	C	0.476	0.282	0.592	4.47E-03
P20908	Collagen alpha-1(V) chain OS=Homo sapiens GN=COL5A1	R	GLLGPKGPPGPPG	P	0.002	0.001	0.297	4.48E-03
P16112	Aggrecan core protein OS=Homo sapiens GN=ACAN	Y	PEAGFGASAAPEASR	E	0.002	0.000	0.173	4.80E-03
P02452	Collagen alpha-1(I) chain OS=Homo sapiens GN=COL1A1	L	GRYYR	T	0.019	0.005	0.244	4.89E-03
P20908	Collagen alpha-1(V) chain OS=Homo sapiens GN=COL5A1	K	GSPGPVGFPGDPGPPGEPGP	A	0.001	0.000	0.417	4.90E-03
P02452	Collagen alpha-1(I) chain OS=Homo sapiens GN=COL1A1	K	GDAGAPGAPGSQGAPGLQGMPGERGAA	G	7.027	1.695	0.241	5.16E-03
P02461	Collagen alpha-1(III) chain OS=Homo sapiens GN=COL3A1	S	DGKPGPPGSQGESGRPGPPGPSGPR	G	0.489	0.048	0.098	5.23E-03
P20908	Collagen alpha-1(V) chain OS=Homo sapiens GN=COL5A1	E	KGAPGEK	G	0.554	0.110	0.198	5.53E-03
P20908	Collagen alpha-1(V) chain OS=Homo sapiens GN=COL5A1	P	AGPVGPPGEDGDK	G	0.025	0.009	0.362	5.80E-03
P49747	Cartilage oligomeric matrix protein OS=Homo sapiens GN=COMP	T	IPEDYETHQLR	Q	0.000	0.000	0.397	5.90E-03
P20908	Collagen alpha-1(V) chain OS=Homo sapiens GN=COL5A1	D	VGQMGPPGPPGPR	G	0.052	0.016	0.307	5.94E-03
P20908	Collagen alpha-1(V) chain OS=Homo sapiens GN=COL5A1	G	LEGPPGKTGPIGPQGAPGKPGPDGLR	G	0.001	0.000	0.244	6.00E-03
P20908	Collagen alpha-1(V) chain OS=Homo sapiens GN=COL5A1	G	RPGPQGPPGPAGEKGAPGEK	G	9.073	0.577	0.064	6.43E-03
P02452	Collagen alpha-1(I) chain OS=Homo sapiens GN=COL1A1	R	GRTGDAGPVGPPGPPGPPGPPGPPSAGFDFSF	L	0.183	0.014	0.075	6.45E-03
P02452	Collagen alpha-1(I) chain OS=Homo sapiens GN=COL1A1	P	QPPQEKAHDGGR	Y	0.256	0.158	0.618	6.50E-03
P02461	Collagen alpha-1(III) chain OS=Homo sapiens GN=COL3A1	G	PPGGSGPAGPPGPQGVK	G	12.081	7.056	0.584	6.51E-03
P20908	Collagen alpha-1(V) chain OS=Homo sapiens GN=COL5A1	R	ITSWPKENPGS	W	3.259	0.457	0.140	6.78E-03
P20908	Collagen alpha-1(V) chain OS=Homo sapiens GN=COL5A1	G	NPGAQGLPGPQGAIGPPGEK	G	29.634	2.149	0.073	6.87E-03
P20908	Collagen alpha-1(V) chain OS=Homo sapiens GN=COL5A1	K	GPPGDDGPKGSPGPVGFPG	D	141.469	15.288	0.108	7.19E-03
P20908	Collagen alpha-1(V) chain OS=Homo sapiens GN=COL5A1	G	DSGPKGEK	G	0.034	0.011	0.336	7.79E-03
P20908	Collagen alpha-1(V) chain OS=Homo sapiens GN=COL5A1	P	PGKTGPIGPQGAPGKPGPDGLR	G	1.420	0.350	0.247	8.22E-03
P02461	Collagen alpha-1(III) chain OS=Homo sapiens GN=COL3A1	G	YQGPPGEPGQAGPSGPPGPPGAIGPSGPAGK	D	0.396	0.150	0.379	8.29E-03
P20908	Collagen alpha-1(V) chain OS=Homo sapiens GN=COL5A1	P	IGPPGPPGLPGPPGPK	G	42.011	1.772	0.042	9.24E-03
P02452	Collagen alpha-1(I) chain OS=Homo sapiens GN=COL1A1	P	GPSGNAGPPGPPGPAGK	E	0.058	0.033	0.577	9.29E-03

P02461	Collagen alpha-1(III) chain OS=Homo sapiens GN=COL3A1	P	PGPPGAIGPSGPAGK	D	1.383	0.820	0.593	9.43E-03
P02452	Collagen alpha-1(I) chain OS=Homo sapiens GN=COL1A1	P	AGERGSPGPAGPK	G	0.095	0.048	0.512	0.010
P20908	Collagen alpha-1(V) chain OS=Homo sapiens GN=COL5A1	G	SPGPDGPPGPMGPPGLPGLKGDSGPK	G	135.593	17.215	0.127	0.011
P20908	Collagen alpha-1(V) chain OS=Homo sapiens GN=COL5A1	G	SPGPDGPPGPMGPPGLPGLK	G	70.744	6.498	0.092	0.011
P02461	Collagen alpha-1(III) chain OS=Homo sapiens GN=COL3A1	R	GSEGSPGHPGQPGPPGPPGA	P	0.093	0.035	0.375	0.011
P02461	Collagen alpha-1(III) chain OS=Homo sapiens GN=COL3A1	R	GSDGQPGPPGPPGTAGFPGS	P	0.046	0.017	0.375	0.011
P20908	Collagen alpha-1(V) chain OS=Homo sapiens GN=COL5A1	G	EPGPSGPPGK	R	31.168	21.112	0.677	0.011
P02452	Collagen alpha-1(I) chain OS=Homo sapiens GN=COL1A1	R	GETGPAGPPGAPGAPGAPGPVGPA	G	0.004	0.001	0.164	0.012
P20908	Collagen alpha-1(V) chain OS=Homo sapiens GN=COL5A1	G	PPGPVGLQGLPGPPGEK	G	5.948	0.226	0.038	0.012
P20908	Collagen alpha-1(V) chain OS=Homo sapiens GN=COL5A1	A	GLEGPPGK	T	0.023	0.012	0.527	0.012
P16112	Aggrecan core protein OS=Homo sapiens GN=ACAN	G	IAEVSGESSR	A	0.004	0.002	0.541	0.013
P20908	Collagen alpha-1(V) chain OS=Homo sapiens GN=COL5A1	P	GRPGPQGPPGPAGEKGAPGEK	G	4.903	0.506	0.103	0.013
P20908	Collagen alpha-1(V) chain OS=Homo sapiens GN=COL5A1	P	NGPQGPTGFPGPKGPPGPPGK	D	4.903	0.506	0.103	0.013
P20908	Collagen alpha-1(V) chain OS=Homo sapiens GN=COL5A1	G	LQGLPGPPGEK	G	1.690	0.834	0.493	0.013
P49747	Cartilage oligomeric matrix protein OS=Homo sapiens GN=COMP		VPDTACVLLLTLAAL	G	4.734	2.264	0.478	0.014
P02452	Collagen alpha-1(I) chain OS=Homo sapiens GN=COL1A1	P	GSPGEQGPSGASGPAGPRGPPGSAGAPGK	D	0.030	0.016	0.550	0.014
P02452	Collagen alpha-1(I) chain OS=Homo sapiens GN=COL1A1	S	GPPGPPGPAGK	E	0.643	0.384	0.598	0.014
P02452	Collagen alpha-1(I) chain OS=Homo sapiens GN=COL1A1	G	ARGPAGPQGPR	G	0.148	0.076	0.512	0.014
P20908	Collagen alpha-1(V) chain OS=Homo sapiens GN=COL5A1	K	GSIGFPGFPGANG	E	0.147	0.076	0.513	0.014
P20908	Collagen alpha-1(V) chain OS=Homo sapiens GN=COL5A1	P	EELTPTPTEAAPMPETSEGAGK	E	0.500	0.071	0.142	0.016
P02461	Collagen alpha-1(III) chain OS=Homo sapiens GN=COL3A1	R	GPTGPIGPPGPAGQP	G	0.311	0.184	0.591	0.016
P20908	Collagen alpha-1(V) chain OS=Homo sapiens GN=COL5A1	G	PTGEPGPSGPPGK	R	1.761	0.422	0.240	0.016
P20908	Collagen alpha-1(V) chain OS=Homo sapiens GN=COL5A1	E	GPPGPPGPAGSPGER	G	23.383	3.460	0.148	0.016
P02452	Collagen alpha-1(I) chain OS=Homo sapiens GN=COL1A1	E	TTGVEGPK	G	0.007	0.004	0.552	0.016
P20908	Collagen alpha-1(V) chain OS=Homo sapiens GN=COL5A1	G	PQGAIGPPGEK	G	1.185	0.666	0.562	0.016
P02452	Collagen alpha-1(I) chain OS=Homo sapiens GN=COL1A1	G	LPGERGRPGAPGPAGAR	G	0.229	0.048	0.210	0.017
P20908	Collagen alpha-1(V) chain OS=Homo sapiens GN=COL5A1	P	AGAAGPIGIPGRPGPQGPPGPAGEK	G	5.529	0.258	0.047	0.017
P02461	Collagen alpha-1(III) chain OS=Homo sapiens GN=COL3A1	K	GAAGPPGPPGAAGTPGLQGMP	G	0.335	0.152	0.454	0.017
P49747	Cartilage oligomeric matrix protein OS=Homo sapiens GN=COMP	D	TTMRGGR	L	0.002	0.001	0.266	0.018
P02461	Collagen alpha-1(III) chain OS=Homo sapiens GN=COL3A1	Q	GPQGPKGDPGPPGIPGR	N	0.017	0.006	0.347	0.018
P02461	Collagen alpha-1(III) chain OS=Homo sapiens GN=COL3A1	K	GDPGPPGIPGRNGDPGIPGQPGSPG	S	2.284	0.664	0.290	0.018
P02461	Collagen alpha-1(III) chain OS=Homo sapiens GN=COL3A1	G	PQGPKGDPGPPGIPGR	N	1833.521	601.482	0.328	0.018
P20908	Collagen alpha-1(V) chain OS=Homo sapiens GN=COL5A1	R	DSFKVYCNFTAGGSTCV	F	0.003	0.001	0.377	0.018
P02461	Collagen alpha-1(III) chain OS=Homo sapiens GN=COL3A1	G	APGLKGENGLPGENGAPGPMGPR	G	5.175	0.876	0.169	0.019
P02461	Collagen alpha-1(III) chain OS=Homo sapiens GN=COL3A1	E	NGSPGAPGAPGHPGPPGPVGPAGK	S	0.005	0.002	0.517	0.019
P02461	Collagen alpha-1(III) chain OS=Homo sapiens GN=COL3A1	P	GYQGPPGEPGQAGPSGPPGPPGAIGPSGPAGK	D	0.003	0.002	0.483	0.020
P02452	Collagen alpha-1(I) chain OS=Homo sapiens GN=COL1A1	K	GAAGEPGKAG	E	0.072	0.036	0.495	0.022
P20908	Collagen alpha-1(V) chain OS=Homo sapiens GN=COL5A1	D	GPAGPPGERGPNGPQGPTGFPGPK	G	90.685	6.319	0.070	0.022
P20908	Collagen alpha-1(V) chain OS=Homo sapiens GN=COL5A1	G	PAGRDGLQGPVGLPGPAGPVGPPGEDGDK	G	0.097	0.030	0.308	0.023
P02452	Collagen alpha-1(I) chain OS=Homo sapiens GN=COL1A1	R	GRTGDAGPVGPPGPPGPPGPPG	P	0.091	0.029	0.324	0.025
P20908	Collagen alpha-1(V) chain OS=Homo sapiens GN=COL5A1	P	GDVGPQGPRGVQGPPGPAGKPGR	R	19998.200	3588.280	0.179	0.025
P02461	Collagen alpha-1(III) chain OS=Homo sapiens GN=COL3A1	N	GSPGAPGAPGHPGPPGPVGPAGK	S	1.094	0.549	0.502	0.027
P20908	Collagen alpha-1(V) chain OS=Homo sapiens GN=COL5A1	V	PGLPGYPGR	Q	0.135	0.096	0.710	0.027
P20908	Collagen alpha-1(V) chain OS=Homo sapiens GN=COL5A1	P	GSPGPDGPPGPMGPPGLPGLK	G	0.037	0.011	0.291	0.027
P02452	Collagen alpha-1(I) chain OS=Homo sapiens GN=COL1A1	G	PPGPPGSPGEQGPSGASGPAGPR	G	0.094	0.058	0.621	0.028
P20908	Collagen alpha-1(V) chain OS=Homo sapiens GN=COL5A1	H	PGPPGPPGPPGEVIQPLPIQASR	T	79.784	7.546	0.095	0.028
P49747	Cartilage oligomeric matrix protein OS=Homo sapiens GN=COMP	R	AFQTVVLDPEG	D	8.553	4.512	0.528	0.029
P07585	Decorin OS=Homo sapiens GN=DCN	K	LTRVPGGLA	E	72.798	13.453	0.185	0.029
P20908	Collagen alpha-1(V) chain OS=Homo sapiens GN=COL5A1	L	EGPPGKTGPIGPQGAPGKPGPDGLR	G	13.412	7.597	0.566	0.030
P20908	Collagen alpha-1(V) chain OS=Homo sapiens GN=COL5A1	K	GEPAIIEPGMLIEGPPGPEGPAG	L	488.968	60.581	0.124	0.031
P02461	Collagen alpha-1(III) chain OS=Homo sapiens GN=COL3A1	P	RGPTGPIGPPGPAGQPGDK	G	1.865	0.784	0.421	0.033
P02452	Collagen alpha-1(I) chain OS=Homo sapiens GN=COL1A1	G	ASGPMGPRGPPGPPGK	N	0.100	0.035	0.348	0.034
P49747	Cartilage oligomeric matrix protein OS=Homo sapiens GN=COMP	T	IPEDYETHQLRQA		0.002	0.001	0.584	0.034
P02452	Collagen alpha-1(I) chain OS=Homo sapiens GN=COL1A1	A	GEPGKAGER	G	0.001	0.000	0.467	0.035
P02452	Collagen alpha-1(I) chain OS=Homo sapiens GN=COL1A1	K	GEPGDAGAKGDAGPPGPAGPAGP	P	1.584	0.926	0.585	0.035
P02461	Collagen alpha-1(III) chain OS=Homo sapiens GN=COL3A1	A	GIPGFPGMK	G	0.010	0.005	0.460	0.036
P20908	Collagen alpha-1(V) chain OS=Homo sapiens GN=COL5A1	R	GETGFQGKTGPPGPP	G	0.027	0.015	0.555	0.036
P02461	Collagen alpha-1(III) chain OS=Homo sapiens GN=COL3A1	P	GVPGAKGEDGK	D	121.284	30.868	0.255	0.038
P02461	Collagen alpha-1(III) chain OS=Homo sapiens GN=COL3A1	G	MPGPRGSPGPQGVK	G	1.052	0.350	0.333	0.038
P20908	Collagen alpha-1(V) chain OS=Homo sapiens GN=COL5A1	P	PGLPGPPGPK	G	5.099	2.369	0.465	0.040
P02461	Collagen alpha-1(III) chain OS=Homo sapiens GN=COL3A1	K	GDPGPPGIPGRNGDPGIPGQPGSPG	S	0.004	0.001	0.266	0.040
P20908	Collagen alpha-1(V) chain OS=Homo sapiens GN=COL5A1	G	LFGQKGDEGPR	G	0.025	0.015	0.608	0.041
P20908	Collagen alpha-1(V) chain OS=Homo sapiens GN=COL5A1	K	GPPGPPGPPGVTGMDGQPGPKG	N	3.006	1.864	0.620	0.043
P02452	Collagen alpha-1(I) chain OS=Homo sapiens GN=COL1A1	T	GPAGRPGEVGPPGPPGPAGEK	G	0.179	0.110	0.617	0.044
P02461	Collagen alpha-1(III) chain OS=Homo sapiens GN=COL3A1	G	MPGPRGSPGPQGVK	G	0.002	0.001	0.408	0.046
P20908	Collagen alpha-1(V) chain OS=Homo sapiens GN=COL5A1	A	IGPPGEKGPLGKPGLPGMPGADGPPGHPGK	E	24.686	7.024	0.285	0.046
P20908	Collagen alpha-1(V) chain OS=Homo sapiens GN=COL5A1	K	GQKGEPAIIEP	G	0.581	0.433	0.746	0.047
P20908	Collagen alpha-1(V) chain OS=Homo sapiens GN=COL5A1	P	GKTGPIGPQGAPGKPGPDGLR	G	0.348	0.092	0.266	0.047
P02461	Collagen alpha-1(III) chain OS=Homo sapiens GN=COL3A1	R	GSPGGPGAAGFPGARGLPGPP	G	0.173	0.095	0.548	0.047
P02452	Collagen alpha-1(I) chain OS=Homo sapiens GN=COL1A1	R	GLTGPIGPPGPAGAPGDKGESGPSGPA	G	3.461	1.471	0.425	0.048
P02452	Collagen alpha-1(I) chain OS=Homo sapiens GN=COL1A1	R	VGPPGPSGNAGPPGPPGPAGKEG	G	1.068	0.336	0.314	0.048
P20908	Collagen alpha-1(V) chain OS=Homo sapiens GN=COL5A1	K	GKLGVPGLP	G	0.241	0.021	0.086	0.048
P02461	Collagen alpha-1(III) chain OS=Homo sapiens GN=COL3A1	G	LPGIAGPRGSPGER	G	0.137	0.079	0.578	0.049
P02452	Collagen alpha-1(I) chain OS=Homo sapiens GN=COL1A1	K	GNSGEPGAP	G	0.598	0.407	0.681	0.049
P02452	Collagen alpha-1(I) chain OS=Homo sapiens GN=COL1A1	L	RGFPGER	G	0.024	0.017	0.686	0.049
P02461	Collagen alpha-1(III) chain OS=Homo sapiens GN=COL3A1	Q	GPQGPKGDPGPPGIPGR	N	113.223	25.338	0.224	0.050
P20908	Collagen alpha-1(V) chain OS=Homo sapiens GN=COL5A1	K	EPTPSKKP	V	0.330	0.238	0.721	0.052
P20908	Collagen alpha-1(V) chain OS=Homo sapiens GN=COL5A1	G	PSGAAGPPGPK	G	0.066	0.045	0.686	0.053
P20908	Collagen alpha-1(V) chain OS=Homo sapiens GN=COL5A1	R	GPSGAPGADGPQGPPGGIGNPGAVGEKGEPGE	A	4.566	0.906	0.199	0.053
P02452	Collagen alpha-1(I) chain OS=Homo sapiens GN=COL1A1	G	AAGRVGPPGPSGNAGPPGPPGPAGK	E	0.146	0.051	0.348	0.054
P20908	Collagen alpha-1(V) chain OS=Homo sapiens GN=COL5A1	G	ADGPQGPPGGIGNPGAVGEK	G	0.001	0.001	0.557	0.055
P02452	Collagen alpha-1(I) chain OS=Homo sapiens GN=COL1A1	R	GEPGPPGPAGFAGPPGADG	Q	0.149	0.099	0.666	0.055
P49747	Cartilage oligomeric matrix protein OS=Homo sapiens GN=COMP	V	GLAFAKANK	Q	66.099	14.851	0.225	0.056
P02461	Collagen alpha-1(III) chain OS=Homo sapiens GN=COL3A1	P	GVSGPK	G	0.009	0.005	0.576	0.056
P07585	Decorin OS=Homo sapiens GN=DCN	I	QLGNYK		0.288	0.159	0.553	0.057
P20908	Collagen alpha-1(V) chain OS=Homo sapiens GN=COL5A1	L	PGEGGPPGPK	G	0.975	0.104	0.106	0.058
P20908	Collagen alpha-1(V) chain OS=Homo sapiens GN=COL5A1	K	GPPGDDGPKGSPGPVGFPG	D	8.001	4.632	0.579	0.059
P20908	Collagen alpha-1(V) chain OS=Homo sapiens GN=COL5A1	G	DPGPSGPPGPPGDDGER	G	0.174	0.062	0.354	0.060
P20908	Collagen alpha-1(V) chain OS=Homo sapiens GN=COL5A1	K	GEPAIIEPGMLIEGPPGPEGPAGL	P	46.766	12.718	0.272	0.061
P02452	Collagen alpha-1(I) chain OS=Homo sapiens GN=COL1A1	R	DGIPGQPGLPGPPGPPGPP	G	62.483	5.116	0.082	0.063
Q06828	Fibromodulin OS=Homo sapiens GN=FMOD	L	DLSYNQLQK	I	0.555	0.415	0.748	0.068
P20908	Collagen alpha-1(V) chain OS=Homo sapiens GN=COL5A1	K	GPPGDDGPKGSPGPVGFPGDPGPP	G	5.740	1.115	0.194	0.069
P02461	Collagen alpha-1(III) chain OS=Homo sapiens GN=COL3A1	D	QASGNVK	K	0.851	0.496	0.583	0.069
P20908	Collagen alpha-1(V) chain OS=Homo sapiens GN=COL5A1	V	PGPKGAK	G	0.102	0.030	0.295	0.070
P02461	Collagen alpha-1(III) chain OS=Homo sapiens GN=COL3A1	Q	GESGRPGPPGPSGPR	G	3.908	2.770	0.709	0.072
P02452	Collagen alpha-1(I) chain OS=Homo sapiens GN=COL1A1	G	PPGPPGPPSAGFDFSFLPQPPQEK	A	0.096	0.063	0.655	0.073
P02452	Collagen alpha-1(I) chain OS=Homo sapiens GN=COL1A1	K	GEPGDAGAKGDAGPPGPAG	P	0.022	0.011	0.486	0.074
P49747	Cartilage oligomeric matrix protein OS=Homo sapiens GN=COMP	R	AFQTVVLDPEGDAQIDP	N	1.158	0.696	0.601	0.075
P02452	Collagen alpha-1(I) chain OS=Homo sapiens GN=COL1A1	G	PQGPGGPPGPK	G	0.021	0.012	0.591	0.075
P02452	Collagen alpha-1(I) chain OS=Homo sapiens GN=COL1A1	G	FQGPPGEPGEPGASGPMGPR	G	0.002	0.001	0.397	0.075
P02452	Collagen alpha-1(I) chain OS=Homo sapiens GN=COL1A1	D	QQTGNLK	K	0.008	0.002	0.279	0.075
P20908	Collagen alpha-1(V) chain OS=Homo sapiens GN=COL5A1	K	EGPPGEKGGQGPPGPQGPIGY	P	0.141	0.073	0.520	0.076
P02452	Collagen alpha-1(I) chain OS=Homo sapiens GN=COL1A1	P	GEAGLPGAK	G	0.079	0.055	0.700	0.076
P20908	Collagen alpha-1(V) chain OS=Homo sapiens GN=COL5A1	G	PSGPIGPPGPPGLPGPPGPK	G	0.018	0.002	0.126	0.077
P02461	Collagen alpha-1(III) chain OS=Homo sapiens GN=COL3A1	K	GEGGPPGVAGPPGGSGP	A	0.008	0.004	0.556	0.077
P16112	Aggrecan core protein OS=Homo sapiens GN=ACAN	Q	TGYPDPSSR	Y	0.004	0.001	0.289	0.078
P07585	Decorin OS=Homo sapiens GN=DCN	K	VPKDLPP	D	20.495	4.776	0.233	0.078

P20908	Collagen alpha-1(V) chain OS=Homo sapiens GN=COL5A1	G	DPGPLGPPGEK	G	4.619	2.983	0.646	0.079
P20908	Collagen alpha-1(V) chain OS=Homo sapiens GN=COL5A1	R	GPSGAPGADGPQGPPGGIGNPGAV	G	0.885	0.676	0.764	0.080
P07585	Decorin OS=Homo sapiens GN=DCN	R	VDAASLKGLNNLA	K	0.032	0.021	0.652	0.082
P02452	Collagen alpha-1(I) chain OS=Homo sapiens GN=COL1A1	R	GETGPAGRPGEVGPPGPPGPAGEKGSPG	A	0.077	0.054	0.706	0.082
P02452	Collagen alpha-1(I) chain OS=Homo sapiens GN=COL1A1	P	GARGPSGPQGPGGPPGPK	G	0.010	0.003	0.326	0.083
P20908	Collagen alpha-1(V) chain OS=Homo sapiens GN=COL5A1	P	PGPPGEK	G	0.048	0.008	0.171	0.084
P02461	Collagen alpha-1(III) chain OS=Homo sapiens GN=COL3A1	A	GPQGPRGDK	G	5.230	1.802	0.345	0.084
P02452	Collagen alpha-1(I) chain OS=Homo sapiens GN=COL1A1	G	DLGAPGPSGAR	G	0.001	0.001	0.653	0.086
P02452	Collagen alpha-1(I) chain OS=Homo sapiens GN=COL1A1	P	PGAPGAPGAPGPVGPAGK	S	0.001	0.001	0.494	0.088
P20908	Collagen alpha-1(V) chain OS=Homo sapiens GN=COL5A1	R	GPSGAPGADGPQGPPGGIGNPGAVGEKGEPGEAGE	P	2.849	1.837	0.645	0.089
P20908	Collagen alpha-1(V) chain OS=Homo sapiens GN=COL5A1	G	PNGDPGPLGPPGEK	G	0.295	0.220	0.746	0.089
P20908	Collagen alpha-1(V) chain OS=Homo sapiens GN=COL5A1	Q	GLPGSPGPDGPPGPMGPPGLPGLKGDSGPK	G	13.121	5.143	0.392	0.090
P20908	Collagen alpha-1(V) chain OS=Homo sapiens GN=COL5A1	P	GPPGEKGK	P	0.003	0.002	0.597	0.092
P02461	Collagen alpha-1(III) chain OS=Homo sapiens GN=COL3A1	A	GPAGAPGPAGSR	G	0.000	0.000	0.405	0.095
P02461	Collagen alpha-1(III) chain OS=Homo sapiens GN=COL3A1	K	GHRGFPGNPGAPGSPGPAGQQGAIGSPG	P	0.143	0.092	0.641	0.097
P20908	Collagen alpha-1(V) chain OS=Homo sapiens GN=COL5A1	R	GIPGPVGEQGLPGSPGPDGPPGPMGPPGLPGLKGDSGP	K	0.096	0.027	0.278	0.097
P02452	Collagen alpha-1(I) chain OS=Homo sapiens GN=COL1A1	G	PIGPPGPAGAPGDK	G	6.131	0.576	0.094	0.097
P20908	Collagen alpha-1(V) chain OS=Homo sapiens GN=COL5A1	R	GPSGAPGADGPQGPPGGIGNPGAVGEKGEP	G	4.571	1.579	0.345	0.101
P20908	Collagen alpha-1(V) chain OS=Homo sapiens GN=COL5A1	R	GPSGAPGADGPQGPPGGIGNPGAVGEKGEPG	E	0.109	0.039	0.359	0.101
P02461	Collagen alpha-1(III) chain OS=Homo sapiens GN=COL3A1	R	GLPGPPGSNGNPGPPGPSGSPGKDGPPGPAG	N	0.094	0.061	0.656	0.103
P02461	Collagen alpha-1(III) chain OS=Homo sapiens GN=COL3A1	P	GMKGHR	G	12.804	3.090	0.241	0.105
P02452	Collagen alpha-1(I) chain OS=Homo sapiens GN=COL1A1	P	PGAPGAPGAPGPVGPAGK	S	0.007	0.004	0.497	0.106
P20908	Collagen alpha-1(V) chain OS=Homo sapiens GN=COL5A1	P	PGPAGKPGR	R	0.675	0.101	0.150	0.107
P20908	Collagen alpha-1(V) chain OS=Homo sapiens GN=COL5A1	G	PPGPAGEKGAPGEK	G	0.698	0.486	0.697	0.107
P20908	Collagen alpha-1(V) chain OS=Homo sapiens GN=COL5A1	P	GPPGPPGVTGMDGQPGPK	G	25.269	0.746	0.030	0.107
P02461	Collagen alpha-1(III) chain OS=Homo sapiens GN=COL3A1	K	GPAGERGAPGPA	G	0.010	0.006	0.567	0.107
Q06828	Fibromodulin OS=Homo sapiens GN=FMOD	R	KVPDGLPS	A	0.205	0.134	0.654	0.108
P02461	Collagen alpha-1(III) chain OS=Homo sapiens GN=COL3A1	R	DGVPGGPGMRGMPGSPGGPGSDGKPGPPGSQGESGRP	G	2.082	1.277	0.613	0.111
P02461	Collagen alpha-1(III) chain OS=Homo sapiens GN=COL3A1	K	GHRGFPGNPGAPGSP	G	0.017	0.011	0.652	0.114
P02452	Collagen alpha-1(I) chain OS=Homo sapiens GN=COL1A1	R	GRTGDAGPVGPPGPPGPPGPPGPPS	A	0.417	0.043	0.103	0.114
P20908	Collagen alpha-1(V) chain OS=Homo sapiens GN=COL5A1	T	GDVGQMGPPGPPGPRGPSGAPGADGPQGPPGGIGNPGAVGEK	G	0.242	0.147	0.606	0.115
P02452	Collagen alpha-1(I) chain OS=Homo sapiens GN=COL1A1	A	TGFPGAAGRVGPPGPSGNAGPPGPPGPAGK	E	0.001	0.000	0.094	0.116
P20908	Collagen alpha-1(V) chain OS=Homo sapiens GN=COL5A1	A	PGADGPQGPPGGIGNPGAVGEK	G	15.589	5.539	0.355	0.117
P02452	Collagen alpha-1(I) chain OS=Homo sapiens GN=COL1A1	A	GPQGPRGDK	G	1.692	0.972	0.574	0.118
P02461	Collagen alpha-1(III) chain OS=Homo sapiens GN=COL3A1	P	PGPAGNTGAPGSPGVSGPK	G	0.244	0.179	0.733	0.118
P02452	Collagen alpha-1(I) chain OS=Homo sapiens GN=COL1A1	R	GFPGLPGPSGEPGKQGPSGA	S	0.010	0.007	0.675	0.122
P02452	Collagen alpha-1(I) chain OS=Homo sapiens GN=COL1A1	T	PGPKGDR	G	0.007	0.003	0.411	0.122
P20908	Collagen alpha-1(V) chain OS=Homo sapiens GN=COL5A1	L	PGADGPPGHPGK	E	1.525	0.789	0.517	0.122
P02461	Collagen alpha-1(III) chain OS=Homo sapiens GN=COL3A1	R	GSDGQPGPPGPPGTAGFPGSPGA	K	0.006	0.003	0.481	0.122
P20908	Collagen alpha-1(V) chain OS=Homo sapiens GN=COL5A1	Q	QGNPGAQGLPGPQGAIGPPGEK	G	0.196	0.146	0.746	0.122
P02461	Collagen alpha-1(III) chain OS=Homo sapiens GN=COL3A1	G	AAGIKGHR	G	0.017	0.007	0.392	0.125
P02461	Collagen alpha-1(III) chain OS=Homo sapiens GN=COL3A1	R	GGAGPPGPEGGKGAAGPPGPPGAAGTP	G	7026.647	3860.739	0.549	0.127
P20908	Collagen alpha-1(V) chain OS=Homo sapiens GN=COL5A1	P	PGPPGEK	G	0.037	0.009	0.243	0.128
P20908	Collagen alpha-1(V) chain OS=Homo sapiens GN=COL5A1	E	SGPSGAAGPPGPKGPPGDDGPK	G	27605.551	13084.574	0.474	0.129
P02452	Collagen alpha-1(I) chain OS=Homo sapiens GN=COL1A1	S	GDRGETGPAG	P	0.032	0.020	0.623	0.130
P02452	Collagen alpha-1(I) chain OS=Homo sapiens GN=COL1A1	R	GPAGPPGRDGIPGQPGLPGPPG	P	0.074	0.022	0.296	0.131
P02452	Collagen alpha-1(I) chain OS=Homo sapiens GN=COL1A1	E	PGASGPMGPRGPPGPPGK	N	37.542	10.828	0.288	0.131
P20908	Collagen alpha-1(V) chain OS=Homo sapiens GN=COL5A1	E	PGEAGEPGLPGEGGPPGPKGER	G	0.146	0.092	0.630	0.131
P07585	Decorin OS=Homo sapiens GN=DCN	K	ATIILLLLAQVSWAGPFQQ	R	17278.746	5300.287	0.307	0.136
P02461	Collagen alpha-1(III) chain OS=Homo sapiens GN=COL3A1	K	NGETGPQGPPGPTGP	G	0.001	0.001	0.704	0.137
P02452	Collagen alpha-1(I) chain OS=Homo sapiens GN=COL1A1	R	GRTGDAGPVGPPGPPGPPGPPGPPS	A	1.015	0.705	0.695	0.138
P02461	Collagen alpha-1(III) chain OS=Homo sapiens GN=COL3A1	P	GNPGAPGSPGPAGQQGAIGSPGPAGPR	G	0.523	0.193	0.369	0.138
P02452	Collagen alpha-1(I) chain OS=Homo sapiens GN=COL1A1	F	SFVDLR	L	0.002	0.001	0.465	0.141
P20908	Collagen alpha-1(V) chain OS=Homo sapiens GN=COL5A1	D	VGQMGPPGPPGPR	G	277.724	39.618	0.143	0.148
P02461	Collagen alpha-1(III) chain OS=Homo sapiens GN=COL3A1	G	PSGPAGK	D	0.056	0.023	0.412	0.148
P02452	Collagen alpha-1(I) chain OS=Homo sapiens GN=COL1A1	D	AGPVGPPGPPGPPGPPGPPSAGFDFSFLPQPPQEK	A	0.608	0.358	0.588	0.151
P02461	Collagen alpha-1(III) chain OS=Homo sapiens GN=COL3A1	K	NGERGGPGGPGPQ	G	0.005	0.003	0.696	0.151
P20908	Collagen alpha-1(V) chain OS=Homo sapiens GN=COL5A1	G	GDGPAGPPGERGPNGPQGPTGFPGPK	G	1.035	0.766	0.740	0.151
P02452	Collagen alpha-1(I) chain OS=Homo sapiens GN=COL1A1	K	QGPSGASGERGPPGPMGPPGLAGPPGESG	R	32.490	3.115	0.096	0.154
P20908	Collagen alpha-1(V) chain OS=Homo sapiens GN=COL5A1	R	GDPGPSGPPGPPGDD	G	1.679	1.237	0.737	0.156
P20908	Collagen alpha-1(V) chain OS=Homo sapiens GN=COL5A1	P	LGKPGLPGMPGADGPPGHPGK	E	7.249	1.494	0.206	0.157
P02452	Collagen alpha-1(I) chain OS=Homo sapiens GN=COL1A1	R	GVPGPPGAVGPAGKDGEAGAQGPPGPAGPA	G	0.209	0.068	0.323	0.158
P02461	Collagen alpha-1(III) chain OS=Homo sapiens GN=COL3A1	P	GENGAPGPMGPR	G	0.145	0.047	0.322	0.158
P20908	Collagen alpha-1(V) chain OS=Homo sapiens GN=COL5A1	R	GPAGAAGPIGIP	G	0.204	0.083	0.407	0.159
P02452	Collagen alpha-1(I) chain OS=Homo sapiens GN=COL1A1	R	GSPGPAGPKGSPGEAGRPGEAGLPG	A	0.067	0.040	0.594	0.159
P20908	Collagen alpha-1(V) chain OS=Homo sapiens GN=COL5A1	P	QGAIGPPGEK	G	0.352	0.255	0.726	0.163
P02452	Collagen alpha-1(I) chain OS=Homo sapiens GN=COL1A1	G	EPGAPGSKGDTGAK	G	3.039	1.998	0.658	0.163
P02461	Collagen alpha-1(III) chain OS=Homo sapiens GN=COL3A1	S	GPAGPAGAPGPAGSR	G	1.839	1.206	0.655	0.164
P02452	Collagen alpha-1(I) chain OS=Homo sapiens GN=COL1A1	G	PPGFPGAVGAKGEAGPQGPR	G	7.460	0.375	0.050	0.165
P02452	Collagen alpha-1(I) chain OS=Homo sapiens GN=COL1A1	G	FSGLQGPPGPPGSPGEQGPSGASGPAGPRGPPGSAGAPGK	D	0.125	0.017	0.133	0.167
P02452	Collagen alpha-1(I) chain OS=Homo sapiens GN=COL1A1	T	GVEGPK	G	0.096	0.072	0.748	0.169
P20908	Collagen alpha-1(V) chain OS=Homo sapiens GN=COL5A1	E	PGEAGEPGLPGEGGPPGPKGER	G	0.016	0.009	0.518	0.170
P02461	Collagen alpha-1(III) chain OS=Homo sapiens GN=COL3A1	A	GAPGAPGGK	G	0.026	0.008	0.324	0.173
P02452	Collagen alpha-1(I) chain OS=Homo sapiens GN=COL1A1	R	GGPGSRGF	P	0.000	0.000	0.130	0.175
P02461	Collagen alpha-1(III) chain OS=Homo sapiens GN=COL3A1	R	GPPGPQGLPGL	A	0.001	0.000	0.360	0.175
P02452	Collagen alpha-1(I) chain OS=Homo sapiens GN=COL1A1	R	DGIPGQPGLPGPPGPPGPPGPPGLGGN	F	0.109	0.016	0.150	0.178
P02452	Collagen alpha-1(I) chain OS=Homo sapiens GN=COL1A1	P	AGFAGPPGADGQPGAK	G	0.027	0.019	0.709	0.181
P02452	Collagen alpha-1(I) chain OS=Homo sapiens GN=COL1A1	F	SGLDGAKGDAGPAGPK	G	0.027	0.019	0.709	0.181
P20908	Collagen alpha-1(V) chain OS=Homo sapiens GN=COL5A1	A	GPIGIPGRPGPQGPPGPAGEKGAPGEK	G	0.034	0.012	0.363	0.183
P02461	Collagen alpha-1(III) chain OS=Homo sapiens GN=COL3A1	P	QYDSYDVK	S	0.078	0.038	0.482	0.185
P02461	Collagen alpha-1(III) chain OS=Homo sapiens GN=COL3A1	K	GDAGAPGAPGGKGDAGAP	G	2.020	1.360	0.673	0.186
P20908	Collagen alpha-1(V) chain OS=Homo sapiens GN=COL5A1	K	GNEGPPGPPGPAGSPGERGPAG	A	0.217	0.120	0.554	0.188
P02452	Collagen alpha-1(I) chain OS=Homo sapiens GN=COL1A1	R	GRPGAPGPAGARGNDGATGAAGPPGPTGPAGPPGFP	G	0.013	0.001	0.076	0.188
P02452	Collagen alpha-1(I) chain OS=Homo sapiens GN=COL1A1	K	GEPGDAGAKGDAGPPGPAGPAGPPGPI	G	4.711	0.756	0.161	0.188
P20908	Collagen alpha-1(V) chain OS=Homo sapiens GN=COL5A1	I	GIPGRPGPQGPPGPAGEK	G	5.352	2.567	0.480	0.188
P02452	Collagen alpha-1(I) chain OS=Homo sapiens GN=COL1A1	G	DDGEAGKPGRPGERGPPGPQGAR	G	2.161	0.270	0.125	0.189
P20908	Collagen alpha-1(V) chain OS=Homo sapiens GN=COL5A1	Q	GITGPSGPIGPPGPPGLPGPPGPK	G	0.001	0.001	0.605	0.190
P20908	Collagen alpha-1(V) chain OS=Homo sapiens GN=COL5A1	G	PKGPPGPPGK	D	0.252	0.157	0.624	0.190
P02452	Collagen alpha-1(I) chain OS=Homo sapiens GN=COL1A1	G	DAGPAGPKGEPGSPGENGAPGQMGPR	G	8.261	0.842	0.102	0.191
P02452	Collagen alpha-1(I) chain OS=Homo sapiens GN=COL1A1	K	GDAGPPGPAGPAGPPGPIGNVG	A	1.210	0.069	0.057	0.197
P02452	Collagen alpha-1(I) chain OS=Homo sapiens GN=COL1A1	V	CPDGSESPTDQETTGVEGPK	G	0.003	0.002	0.744	0.197
P20908	Collagen alpha-1(V) chain OS=Homo sapiens GN=COL5A1	R	DGLQGPVGLPGPAGPVGPPGEDGDKGEI	G	2.679	1.659	0.619	0.199
P02452	Collagen alpha-1(I) chain OS=Homo sapiens GN=COL1A1	E	TGPAGPPGAPGAPGAPGPVGPAGKSGDR	G	6.330	1.220	0.193	0.201
P20908	Collagen alpha-1(V) chain OS=Homo sapiens GN=COL5A1	A	QLSAPTK	Q	0.001	0.001	0.666	0.202
P02452	Collagen alpha-1(I) chain OS=Homo sapiens GN=COL1A1	R	GEPGPPGPAGAAGPAGNPGADGQPGAKGANGA	P	0.530	0.068	0.129	0.204
P20908	Collagen alpha-1(V) chain OS=Homo sapiens GN=COL5A1	T	GEPGPSGPPGK	R	4.074	3.102	0.761	0.204
P02452	Collagen alpha-1(I) chain OS=Homo sapiens GN=COL1A1	R	GSEGPQGV	R	0.000	0.000	0.292	0.204
P20908	Collagen alpha-1(V) chain OS=Homo sapiens GN=COL5A1	G	EPGEAGEPGLPGEGGPPGPKGER	G	4309.961	1445.738	0.335	0.206
P20908	Collagen alpha-1(V) chain OS=Homo sapiens GN=COL5A1	G	PVGPPGSGGLK	G	0.043	0.031	0.704	0.207
P02461	Collagen alpha-1(III) chain OS=Homo sapiens GN=COL3A1	P	GINGSPGGK	G	0.001	0.000	0.180	0.208
P02461	Collagen alpha-1(III) chain OS=Homo sapiens GN=COL3A1	Q	GPPGAPGPLGIAGITGARGLAGPPGMPGPR	G	0.263	0.203	0.774	0.209
P02452	Collagen alpha-1(I) chain OS=Homo sapiens GN=COL1A1	R	GETGPAGPPGAPGAPGAPGPVG	P	2.942	0.515	0.175	0.210
P07585	Decorin OS=Homo sapiens GN=DCN	Y	LSKNQLK	E	0.135	0.075	0.554	0.211
P02452	Collagen alpha-1(I) chain OS=Homo sapiens GN=COL1A1	R	GEPGPPGPAGAAGP	A	0.391	0.059	0.152	0.211
P02461	Collagen alpha-1(III) chain OS=Homo sapiens GN=COL3A1	P	PGPPGAIGPSGPAGK	D	0.012	0.010	0.786	0.212
P02452	Collagen alpha-1(I) chain OS=Homo sapiens GN=COL1A1	M	GPRGPPGPPGK	N	0.262	0.161	0.614	0.215
P02452	Collagen alpha-1(I) chain OS=Homo sapiens GN=COL1A1	E	PGASGPMGPR	G	0.045	0.034	0.758	0.215

P20908	Collagen alpha-1(V) chain OS=Homo sapiens GN=COL5A1	V	VGPQGPR	G	0.008	0.004	0.530	0.216
P16112	Aggrecan core protein OS=Homo sapiens GN=ACAN	K	DSSPGVRTYG	V	0.000	0.000	0.464	0.217
P02461	Collagen alpha-1(III) chain OS=Homo sapiens GN=COL3A1	R	GPVGPSGPP	G	0.015	0.009	0.571	0.218
P02452	Collagen alpha-1(I) chain OS=Homo sapiens GN=COL1A1	R	GQAGVMGFPGPKGA	A	0.344	0.260	0.754	0.220
P20908	Collagen alpha-1(V) chain OS=Homo sapiens GN=COL5A1	G	PQGPPGGIGNPGAVGEKGEPGEAGEPGLPGEGGPPGPK	G	40.003	15.172	0.379	0.222
P02452	Collagen alpha-1(I) chain OS=Homo sapiens GN=COL1A1	R	GAPGDRGEPGPPGPAGFAGPPGA	D	0.013	0.010	0.759	0.222
P20908	Collagen alpha-1(V) chain OS=Homo sapiens GN=COL5A1	G	PAGPVGPPGEDGDK	G	10.526	6.079	0.577	0.222
P20908	Collagen alpha-1(V) chain OS=Homo sapiens GN=COL5A1	K	GDRGLPGPQ	G	0.000	0.000	0.190	0.223
P02452	Collagen alpha-1(I) chain OS=Homo sapiens GN=COL1A1	K	GDAGPAGPKGEPGSPGENGAPGQ	M	6.358	1.007	0.158	0.224
P02452	Collagen alpha-1(I) chain OS=Homo sapiens GN=COL1A1	S	PGSPGPDGK	T	0.007	0.002	0.257	0.225
P02461	Collagen alpha-1(III) chain OS=Homo sapiens GN=COL3A1	R	GLAGPPGM	P	0.007	0.002	0.369	0.225
P02452	Collagen alpha-1(I) chain OS=Homo sapiens GN=COL1A1	P	PGPPGPAGKEGGK	G	0.791	0.627	0.794	0.226
P02461	Collagen alpha-1(III) chain OS=Homo sapiens GN=COL3A1	K	LMGSNEGEFKAEGN	S	400.817	303.134	0.756	0.228
P02452	Collagen alpha-1(I) chain OS=Homo sapiens GN=COL1A1	K	GEPGSPGENGAPGQMGP	R	0.008	0.005	0.662	0.228
P02452	Collagen alpha-1(I) chain OS=Homo sapiens GN=COL1A1	P	GPPGAPGPQGFQGPPGEPGEPGASGPMGPR	G	1.699	0.978	0.576	0.229
P02461	Collagen alpha-1(III) chain OS=Homo sapiens GN=COL3A1	P	GPPGPQGL	Q	0.003	0.001	0.349	0.231
P02461	Collagen alpha-1(III) chain OS=Homo sapiens GN=COL3A1	K	GSPGAQGPPGA	P	0.052	0.028	0.544	0.234
P02452	Collagen alpha-1(I) chain OS=Homo sapiens GN=COL1A1	P	TQPSVAQKNWYISK	N	0.004	0.002	0.494	0.236
P02452	Collagen alpha-1(I) chain OS=Homo sapiens GN=COL1A1	G	AEVPEGECCPVCPDGSESPTDQETTGVEGPKGDTGPR	G	23.321	3.351	0.144	0.238
P02461	Collagen alpha-1(III) chain OS=Homo sapiens GN=COL3A1	R	GFPGNPGAPGS	P	0.279	0.175	0.627	0.239
P02461	Collagen alpha-1(III) chain OS=Homo sapiens GN=COL3A1	P	GEPGANGLPGAAGER	G	0.001	0.001	0.818	0.243
P20908	Collagen alpha-1(V) chain OS=Homo sapiens GN=COL5A1	K	GPQGPAGRDGLQGPVGLPGPAGPVGP	P	57.336	2.884	0.050	0.244
P02461	Collagen alpha-1(III) chain OS=Homo sapiens GN=COL3A1	A	GPPGPEGGK	G	0.176	0.057	0.324	0.248
P02452	Collagen alpha-1(I) chain OS=Homo sapiens GN=COL1A1	R	GRPGAPGPAG	A	0.000	0.000	0.171	0.250
P02452	Collagen alpha-1(I) chain OS=Homo sapiens GN=COL1A1	G	LPGPIGPPGPR	G	0.457	0.140	0.308	0.252
P02461	Collagen alpha-1(III) chain OS=Homo sapiens GN=COL3A1	R	GLPGPPGSNGNPGPPGPSGSPGKDGPPGPAG	N	1.049	0.692	0.660	0.255
P02461	Collagen alpha-1(III) chain OS=Homo sapiens GN=COL3A1	R	GEPGPQGHAGAQGPPGPPGI	N	0.009	0.006	0.682	0.255
P02461	Collagen alpha-1(III) chain OS=Homo sapiens GN=COL3A1	N	DGARGSDGQPGPPGPPGTAGFPGSPGAK	G	0.066	0.043	0.645	0.255
P16112	Aggrecan core protein OS=Homo sapiens GN=ACAN	P	FTCKK	G	0.055	0.016	0.286	0.261
P02452	Collagen alpha-1(I) chain OS=Homo sapiens GN=COL1A1	K	GDAGPPGPAGPAGPPGPIGNV	G	0.066	0.004	0.063	0.262
P20908	Collagen alpha-1(V) chain OS=Homo sapiens GN=COL5A1	G	PPGEQGLPGLAGK	E	0.013	0.009	0.684	0.263
P20908	Collagen alpha-1(V) chain OS=Homo sapiens GN=COL5A1	K	ARSALRPGAP	L	2.086	0.166	0.079	0.265
P20908	Collagen alpha-1(V) chain OS=Homo sapiens GN=COL5A1	K	GNEGPPGPPGPAGSPGERGPAGAAGPIGIPGRPGPQGPP	G	54.489	29.195	0.536	0.266
P02452	Collagen alpha-1(I) chain OS=Homo sapiens GN=COL1A1	K	GDAGPPGPAGPA	G	0.050	0.003	0.064	0.270
P02452	Collagen alpha-1(I) chain OS=Homo sapiens GN=COL1A1	S	YGYDEKSTGGISVPGPMGPSGPR	G	0.019	0.003	0.173	0.271
P20908	Collagen alpha-1(V) chain OS=Homo sapiens GN=COL5A1	R	GVQGPPGPA	G	0.018	0.006	0.350	0.272
P20908	Collagen alpha-1(V) chain OS=Homo sapiens GN=COL5A1	K	GPPGPPGPPGVTGMDG	Q	0.000	0.000	0.167	0.275
P02461	Collagen alpha-1(III) chain OS=Homo sapiens GN=COL3A1	K	GETGAPGLKGENGLPGENGAPGP	M	0.119	0.091	0.766	0.275
P02452	Collagen alpha-1(I) chain OS=Homo sapiens GN=COL1A1	K	GEPGPVGVQG	P	0.002	0.001	0.513	0.277
P02452	Collagen alpha-1(I) chain OS=Homo sapiens GN=COL1A1	G	PAGNPGADGQPGAK	G	0.004	0.002	0.628	0.278
P20908	Collagen alpha-1(V) chain OS=Homo sapiens GN=COL5A1	E	KGAPGEK	G	0.831	0.552	0.664	0.279
P02461	Collagen alpha-1(III) chain OS=Homo sapiens GN=COL3A1	K	GEVGPAGSPGS	N	0.001	0.000	0.422	0.281
P20908	Collagen alpha-1(V) chain OS=Homo sapiens GN=COL5A1	P	PGPPGPAGSPGER	G	2.733	2.278	0.834	0.282
P02452	Collagen alpha-1(I) chain OS=Homo sapiens GN=COL1A1	G	ADGSPGKDGVR	G	0.000	0.000	0.427	0.282
P02461	Collagen alpha-1(III) chain OS=Homo sapiens GN=COL3A1	G	PPGPSGSPGK	D	0.000	0.000	0.195	0.282
P02461	Collagen alpha-1(III) chain OS=Homo sapiens GN=COL3A1	G	PPGKNGETGPQGPPGPTGPGGDK	G	0.385	0.245	0.635	0.283
P02461	Collagen alpha-1(III) chain OS=Homo sapiens GN=COL3A1	G	APGPQGPR	G	0.000	0.000	0.641	0.285
P02452	Collagen alpha-1(I) chain OS=Homo sapiens GN=COL1A1	S	GVQGPPGP	P	0.006	0.004	0.742	0.285
P02452	Collagen alpha-1(I) chain OS=Homo sapiens GN=COL1A1	P	PGSPGEQGPSGASGPAGPRGPPGSAGAPGK	D	0.042	0.001	0.020	0.286
P02461	Collagen alpha-1(III) chain OS=Homo sapiens GN=COL3A1	M	GPRGAPGER	G	0.671	0.137	0.205	0.291
P02461	Collagen alpha-1(III) chain OS=Homo sapiens GN=COL3A1	R	GPAGPNGIPG	E	0.002	0.001	0.403	0.292
P02461	Collagen alpha-1(III) chain OS=Homo sapiens GN=COL3A1	G	PKGDAGAPGAPGGK	G	0.830	0.031	0.037	0.293
P02452	Collagen alpha-1(I) chain OS=Homo sapiens GN=COL1A1	D	DGEAGKPGRPGERGPPGPQGAR	G	0.581	0.067	0.116	0.296
P02452	Collagen alpha-1(I) chain OS=Homo sapiens GN=COL1A1	A	DGVAGPKGPAGER	G	0.008	0.007	0.794	0.297
P02452	Collagen alpha-1(I) chain OS=Homo sapiens GN=COL1A1	P	KGDTGPR	G	0.016	0.010	0.623	0.297
P02452	Collagen alpha-1(I) chain OS=Homo sapiens GN=COL1A1	G	ASGPAGPR	G	0.001	0.000	0.144	0.297
P20908	Collagen alpha-1(V) chain OS=Homo sapiens GN=COL5A1	P	GPSGPPGKR	G	0.460	0.322	0.700	0.297
P02452	Collagen alpha-1(I) chain OS=Homo sapiens GN=COL1A1	G	EPGSPGENGAPGQMGPRGLPGER	G	0.115	0.064	0.556	0.298
P49747	Cartilage oligomeric matrix protein OS=Homo sapiens GN=COMP	R	VPNSDQKDSD	G	1.890	0.751	0.397	0.298
P02452	Collagen alpha-1(I) chain OS=Homo sapiens GN=COL1A1	R	GPPGPMGPPGLAGPPG	E	0.086	0.005	0.063	0.299
P02452	Collagen alpha-1(I) chain OS=Homo sapiens GN=COL1A1	A	PGPVGPAGK	S	0.001	0.000	0.441	0.300
P20908	Collagen alpha-1(V) chain OS=Homo sapiens GN=COL5A1	L	QGLPGPPGEK	G	0.000	0.000	0.727	0.302
P02452	Collagen alpha-1(I) chain OS=Homo sapiens GN=COL1A1	G	PPGPMGPPGLAGPPGESGR	E	0.268	0.021	0.078	0.302
P02452	Collagen alpha-1(I) chain OS=Homo sapiens GN=COL1A1	G	PPGPMGPPGLAGPPGESGR	E	0.102	0.010	0.099	0.303
P02461	Collagen alpha-1(III) chain OS=Homo sapiens GN=COL3A1	P	PGPQGPPGK	D	0.003	0.001	0.482	0.304
P16112	Aggrecan core protein OS=Homo sapiens GN=ACAN	E	TSAYPEAGFGASAAPEASR	E	0.024	0.002	0.069	0.307
P02461	Collagen alpha-1(III) chain OS=Homo sapiens GN=COL3A1	A	PGPVGPAGK	S	0.000	0.000	0.472	0.308
P02452	Collagen alpha-1(I) chain OS=Homo sapiens GN=COL1A1	G	PVGVQGPPGPAGEEGKR	G	0.070	0.051	0.731	0.309
Q06828	Fibromodulin OS=Homo sapiens GN=FMOD	R	HLERLY	L	0.181	0.074	0.408	0.309
P20908	Collagen alpha-1(V) chain OS=Homo sapiens GN=COL5A1	Q	RGPTGPR	G	0.000	0.000	0.322	0.309
P20908	Collagen alpha-1(V) chain OS=Homo sapiens GN=COL5A1	K	GSPGPVGFPGDPGPPGEPGPAGQDGPP	G	0.729	0.421	0.577	0.312
P20908	Collagen alpha-1(V) chain OS=Homo sapiens GN=COL5A1	G	PTGPQGPIGQPGPSGADGEPGPR	G	1.757	0.647	0.368	0.315
P20908	Collagen alpha-1(V) chain OS=Homo sapiens GN=COL5A1	K	LLSYVDAEGNPVGVVQMT	F	15.885	5.984	0.377	0.315
P16112	Aggrecan core protein OS=Homo sapiens GN=ACAN	E	VSGESSR	A	0.000	0.000	0.552	0.316
P20908	Collagen alpha-1(V) chain OS=Homo sapiens GN=COL5A1	T	SSPSEIGPGMPANQDTIYEGIGGPR	G	53.432	5.002	0.094	0.318
P20908	Collagen alpha-1(V) chain OS=Homo sapiens GN=COL5A1	G	PKGPPGPPGK	D	241.596	149.194	0.618	0.319
P20908	Collagen alpha-1(V) chain OS=Homo sapiens GN=COL5A1	A	PGPPGPR	G	0.021	0.014	0.661	0.324
P20908	Collagen alpha-1(V) chain OS=Homo sapiens GN=COL5A1	K	GEQGITGPSGPIGP	P	0.093	0.068	0.732	0.325
P02461	Collagen alpha-1(III) chain OS=Homo sapiens GN=COL3A1	P	PGPSGPR	G	0.000	0.000	0.432	0.326
P20908	Collagen alpha-1(V) chain OS=Homo sapiens GN=COL5A1	G	SPGPDGPPGPMGPPGLPGLKGDSGPK	G	1.383	0.878	0.635	0.326
P02452	Collagen alpha-1(I) chain OS=Homo sapiens GN=COL1A1	G	SRGFPGADGVAGPK	G	0.022	0.015	0.694	0.328
P02452	Collagen alpha-1(I) chain OS=Homo sapiens GN=COL1A1	G	DAGPAGPKGEPGSPGENGAPGQMGPR	G	1.004	0.094	0.094	0.329
P49747	Cartilage oligomeric matrix protein OS=Homo sapiens GN=COMP	R	VRFYE	G	0.000	0.000	0.598	0.330
P02452	Collagen alpha-1(I) chain OS=Homo sapiens GN=COL1A1	P	GEPGASGPMGPRGPPGPPGK	N	0.057	0.041	0.719	0.331
P02461	Collagen alpha-1(III) chain OS=Homo sapiens GN=COL3A1	R	GLPGPPGSNGNPGPPGPSG	S	0.374	0.272	0.728	0.331
P20908	Collagen alpha-1(V) chain OS=Homo sapiens GN=COL5A1	L	PGEKGHR	G	0.002	0.001	0.592	0.334
P20908	Collagen alpha-1(V) chain OS=Homo sapiens GN=COL5A1	K	GNEGPPGPPGPAGSPG	E	0.128	0.056	0.437	0.335
P20908	Collagen alpha-1(V) chain OS=Homo sapiens GN=COL5A1	P	GEGGPPGPK	G	0.014	0.001	0.047	0.336
P02452	Collagen alpha-1(I) chain OS=Homo sapiens GN=COL1A1	K	GPRGETGP	A	0.003	0.001	0.276	0.345
P02461	Collagen alpha-1(III) chain OS=Homo sapiens GN=COL3A1	D	GNPGSDGLPGR	D	0.158	0.132	0.836	0.347
P20908	Collagen alpha-1(V) chain OS=Homo sapiens GN=COL5A1	P	GPKGPPGDDGPK	G	0.013	0.010	0.746	0.349
P02461	Collagen alpha-1(III) chain OS=Homo sapiens GN=COL3A1	G	APGPLGIAGITGARGLAGPPGMPGPR	G	0.018	0.014	0.774	0.351
P02452	Collagen alpha-1(I) chain OS=Homo sapiens GN=COL1A1	G	PPGPAGQDGRPGPPGPPGAR	G	0.001	0.000	0.313	0.351
P20908	Collagen alpha-1(V) chain OS=Homo sapiens GN=COL5A1	R	GPPGRPGLPGADGLPGP	P	0.019	0.014	0.740	0.351
P20908	Collagen alpha-1(V) chain OS=Homo sapiens GN=COL5A1	P	GVVGPQGPTGETGPMGERGHPGPPGPPGEQGLPGLAGK	E	0.007	0.005	0.747	0.352
P20908	Collagen alpha-1(V) chain OS=Homo sapiens GN=COL5A1	I	ALSVHKK	N	0.001	0.000	0.148	0.353
P02452	Collagen alpha-1(I) chain OS=Homo sapiens GN=COL1A1	R	GPPGPMGPPGLAGPPGESG	R	0.086	0.020	0.232	0.356
P20908	Collagen alpha-1(V) chain OS=Homo sapiens GN=COL5A1	P	GRPGPQGPPGPAGEK	G	0.174	0.141	0.813	0.362
P16112	Aggrecan core protein OS=Homo sapiens GN=ACAN	R	TLVESVTQAP	T	0.049	0.023	0.468	0.363
P02461	Collagen alpha-1(III) chain OS=Homo sapiens GN=COL3A1	P	PGPTGPGGDK	G	0.031	0.022	0.723	0.363
P02461	Collagen alpha-1(III) chain OS=Homo sapiens GN=COL3A1	G	APGPAGPR	G	0.005	0.003	0.644	0.366
P02452	Collagen alpha-1(I) chain OS=Homo sapiens GN=COL1A1	K	STGGISVPGPMGPSGPRGLPGP	P	0.206	0.113	0.549	0.368
P02461	Collagen alpha-1(III) chain OS=Homo sapiens GN=COL3A1	G	APGHPGPPGPVGPAGK	S	13.587	7.834	0.577	0.369
P20908	Collagen alpha-1(V) chain OS=Homo sapiens GN=COL5A1	R	GGPNGDPGPLGPP	G	0.032	0.023	0.737	0.371
P02452	Collagen alpha-1(I) chain OS=Homo sapiens GN=COL1A1	P	PGSPGEQGPSGASGPAGPR	G	0.000	0.000	0.594	0.371
P20908	Collagen alpha-1(V) chain OS=Homo sapiens GN=COL5A1	G	RPGPQGPPGPAGEKGAPGEK	G	0.256	0.220	0.859	0.374
P20908	Collagen alpha-1(V) chain OS=Homo sapiens GN=COL5A1	K	GEIGEPGQ	K	0.000	0.000	0.424	0.375

P20908	Collagen alpha-1(V) chain OS=Homo sapiens GN=COL5A1	K	GNEGPPGPPGPAGSPGERGPAGAAGPIGIP	G	1.055	0.107	0.102	0.377
P02452	Collagen alpha-1(I) chain OS=Homo sapiens GN=COL1A1	T	GPAGPPGAPGAPGAPGPVGPAGKSGDR	G	0.015	0.005	0.310	0.380
P02461	Collagen alpha-1(III) chain OS=Homo sapiens GN=COL3A1	P	GAPGLMGAR	G	0.001	0.000	0.662	0.382
P16112	Aggrecan core protein OS=Homo sapiens GN=ACAN	G	AGEISGLPSSELDISGR	A	0.068	0.040	0.586	0.384
P20908	Collagen alpha-1(V) chain OS=Homo sapiens GN=COL5A1	D	GLPGPPGTMLMLPFRFGGGGDAGSK	G	3.886	2.168	0.558	0.387
P02452	Collagen alpha-1(I) chain OS=Homo sapiens GN=COL1A1	P	SGNAGPPGPPGPAGK	E	0.010	0.007	0.666	0.389
P20908	Collagen alpha-1(V) chain OS=Homo sapiens GN=COL5A1	K	GEPGDVGPQGPRGVQGP	P	11.076	8.012	0.723	0.389
P16112	Aggrecan core protein OS=Homo sapiens GN=ACAN	V	RYPISK	A	0.000	0.000	0.413	0.389
P20908	Collagen alpha-1(V) chain OS=Homo sapiens GN=COL5A1	E	RGPPGPA	G	0.004	0.001	0.413	0.393
P02452	Collagen alpha-1(I) chain OS=Homo sapiens GN=COL1A1	P	GGPPGPKGNSGEPGAPGSK	G	0.001	0.000	0.121	0.394
P02461	Collagen alpha-1(III) chain OS=Homo sapiens GN=COL3A1	Q	GPPGAPGPLGIAGITGARGLAGPPGMPGPR	G	0.048	0.037	0.764	0.394
P02461	Collagen alpha-1(III) chain OS=Homo sapiens GN=COL3A1	R	GAPGEKGEGGPPGVA	G	1.070	0.567	0.530	0.395
P02461	Collagen alpha-1(III) chain OS=Homo sapiens GN=COL3A1	G	PPGMPGPR	G	0.002	0.000	0.048	0.399
P20908	Collagen alpha-1(V) chain OS=Homo sapiens GN=COL5A1	L	PGEKGHR	G	0.009	0.005	0.563	0.401
P02452	Collagen alpha-1(I) chain OS=Homo sapiens GN=COL1A1	G	PAGPQGPR	G	0.000	0.000	0.717	0.401
P20908	Collagen alpha-1(V) chain OS=Homo sapiens GN=COL5A1	R	GFPGPPGPVGLQGLPGPPGEKGET	G	5.307	4.256	0.802	0.403
P20908	Collagen alpha-1(V) chain OS=Homo sapiens GN=COL5A1	L	RGFPGDR	G	0.002	0.001	0.415	0.404
P20908	Collagen alpha-1(V) chain OS=Homo sapiens GN=COL5A1	D	DGEPGQTGSPGPTGEPGPSGPPGKR	G	288.511	170.792	0.592	0.405
P02452	Collagen alpha-1(I) chain OS=Homo sapiens GN=COL1A1	R	GFSGLQGPPGPPGSPGEQGPSGA	S	0.011	0.006	0.605	0.406
P20908	Collagen alpha-1(V) chain OS=Homo sapiens GN=COL5A1	P	GPPGPPGEQGLPGLAGK	E	0.036	0.024	0.672	0.410
P20908	Collagen alpha-1(V) chain OS=Homo sapiens GN=COL5A1	R	GFPGPPGPVGLQGLPGPPGEKGET	G	1.341	1.092	0.814	0.411
P02452	Collagen alpha-1(I) chain OS=Homo sapiens GN=COL1A1	G	PAGPTGPVGPVGARGPAGPQGPR	G	0.001	0.000	0.766	0.418
P20908	Collagen alpha-1(V) chain OS=Homo sapiens GN=COL5A1	Q	GLPGPPGEK	G	0.002	0.001	0.629	0.419
P02461	Collagen alpha-1(III) chain OS=Homo sapiens GN=COL3A1	K	GDAGAPGAPGGKG	D	0.504	0.425	0.843	0.422
P02452	Collagen alpha-1(I) chain OS=Homo sapiens GN=COL1A1	R	GPAGPPGRDGIPGQPGLPGPPGPPGPPGPP	G	4.826	3.726	0.772	0.422
P02452	Collagen alpha-1(I) chain OS=Homo sapiens GN=COL1A1	G	PRGANGAPGNDGAK	G	0.077	0.058	0.759	0.424
P20908	Collagen alpha-1(V) chain OS=Homo sapiens GN=COL5A1	G	PPGHPGK	E	0.052	0.035	0.666	0.426
P02461	Collagen alpha-1(III) chain OS=Homo sapiens GN=COL3A1	L	KFCHPELK	S	0.987	0.772	0.783	0.426
P20908	Collagen alpha-1(V) chain OS=Homo sapiens GN=COL5A1	D	GLQGPVGLPGPAGPVGPPGEDGDK	G	0.173	0.111	0.644	0.427
P20908	Collagen alpha-1(V) chain OS=Homo sapiens GN=COL5A1	G	PPGLPGPPGPKGAK	G	0.138	0.102	0.745	0.429
P20908	Collagen alpha-1(V) chain OS=Homo sapiens GN=COL5A1	K	GEPAIIEPGMLIEGPPGPEGPAG	L	15.328	10.755	0.702	0.432
P20908	Collagen alpha-1(V) chain OS=Homo sapiens GN=COL5A1	K	GEAGHPGPPGPPGPPGEVIQPL	P	6.141	3.999	0.651	0.437
P20908	Collagen alpha-1(V) chain OS=Homo sapiens GN=COL5A1	P	GPAGKPGR	R	0.004	0.002	0.643	0.444
P02452	Collagen alpha-1(I) chain OS=Homo sapiens GN=COL1A1	R	DGIPGQPGLPG	P	0.001	0.000	0.743	0.444
P20908	Collagen alpha-1(V) chain OS=Homo sapiens GN=COL5A1	R	GVQGPPGPAG	P	0.000	0.000	0.450	0.446
P02461	Collagen alpha-1(III) chain OS=Homo sapiens GN=COL3A1	D	GVPGKDGPR	G	0.114	0.048	0.417	0.448
P02461	Collagen alpha-1(III) chain OS=Homo sapiens GN=COL3A1	E	PGKNGAK	G	0.006	0.002	0.410	0.449
P20908	Collagen alpha-1(V) chain OS=Homo sapiens GN=COL5A1	K	GEQGITGPSGPIGPPGPP	G	40.017	35.001	0.875	0.449
P20908	Collagen alpha-1(V) chain OS=Homo sapiens GN=COL5A1	K	GEPGEAGEPGLPGEGGPPGPKGE	R	337.154	229.672	0.681	0.451
P02452	Collagen alpha-1(I) chain OS=Homo sapiens GN=COL1A1	P	PGPPGPAGKEGGK	G	0.021	0.018	0.874	0.451
P20908	Collagen alpha-1(V) chain OS=Homo sapiens GN=COL5A1	G	PSGAAGPPGPK	G	1.262	1.033	0.819	0.454
P02452	Collagen alpha-1(I) chain OS=Homo sapiens GN=COL1A1	P	GAPGPVGPAGK	S	0.062	0.047	0.765	0.456
P20908	Collagen alpha-1(V) chain OS=Homo sapiens GN=COL5A1	Q	GPTGFPGPK	G	0.006	0.004	0.654	0.457
P20908	Collagen alpha-1(V) chain OS=Homo sapiens GN=COL5A1	V	GPPGSGGLKGEPGDVGPQGPR	G	195.422	118.764	0.608	0.457
P20908	Collagen alpha-1(V) chain OS=Homo sapiens GN=COL5A1	E	RGPPGPAG	S	0.006	0.004	0.719	0.460
P20908	Collagen alpha-1(V) chain OS=Homo sapiens GN=COL5A1	K	GPPGPPGPPGVTGMDGQPGPKGNVGPQG	E	0.001	0.001	0.752	0.463
P02461	Collagen alpha-1(III) chain OS=Homo sapiens GN=COL3A1	K	GDAGAPGAPGGKG	D	0.016	0.014	0.872	0.468
P20908	Collagen alpha-1(V) chain OS=Homo sapiens GN=COL5A1	E	PGPSGPPGKR	G	0.001	0.001	0.810	0.470
P02452	Collagen alpha-1(I) chain OS=Homo sapiens GN=COL1A1	D	PGGPPGPK	G	0.032	0.027	0.854	0.471
P07585	Decorin OS=Homo sapiens GN=DCN	N	KITEIK	D	0.024	0.008	0.348	0.473
P02461	Collagen alpha-1(III) chain OS=Homo sapiens GN=COL3A1	S	GPPGKDGTSGHPGPIGPPGPR	G	189.850	126.610	0.667	0.474
P02461	Collagen alpha-1(III) chain OS=Homo sapiens GN=COL3A1	R	GENGSPGAPGAPGHPGPPGPVGP	A	0.035	0.027	0.779	0.476
P02452	Collagen alpha-1(I) chain OS=Homo sapiens GN=COL1A1	K	GLTGSPGSPG	P	0.004	0.002	0.643	0.476
P20908	Collagen alpha-1(V) chain OS=Homo sapiens GN=COL5A1	F	PGANGEK	G	0.003	0.002	0.765	0.476
P02452	Collagen alpha-1(I) chain OS=Homo sapiens GN=COL1A1	R	GANGAPGNDGAKGDAGAPGAPGSQGAP	G	0.322	0.263	0.818	0.478
P02461	Collagen alpha-1(III) chain OS=Homo sapiens GN=COL3A1	G	PPGPTGPGGDK	G	0.002	0.001	0.809	0.480
P02461	Collagen alpha-1(III) chain OS=Homo sapiens GN=COL3A1	K	SGVAVGGLAGYPGPAGPPGPPGPPGTSGHPGSP	G	0.003	0.002	0.531	0.481
P20908	Collagen alpha-1(V) chain OS=Homo sapiens GN=COL5A1	S	GAAGPPGPK	G	0.032	0.021	0.637	0.483
P07585	Decorin OS=Homo sapiens GN=DCN	L	PEKMPK	T	0.050	0.032	0.640	0.484
P02461	Collagen alpha-1(III) chain OS=Homo sapiens GN=COL3A1	E	KGEPGPR	G	0.001	0.001	0.652	0.484
P02452	Collagen alpha-1(I) chain OS=Homo sapiens GN=COL1A1	E	PGPPGPAGFAGPPGADGQPGAK	G	4.000	2.379	0.595	0.485
P02452	Collagen alpha-1(I) chain OS=Homo sapiens GN=COL1A1	E	PGEPGASGPMGPR	G	0.002	0.001	0.472	0.491
P20908	Collagen alpha-1(V) chain OS=Homo sapiens GN=COL5A1	A	QPADLLK	V	0.000	0.000	0.817	0.492
P02452	Collagen alpha-1(I) chain OS=Homo sapiens GN=COL1A1	G	EPGPPGPAGAAGPAGNPGADGQPGAKGANGAPGIAGAPGFPGAR	G	152.262	121.483	0.798	0.493
P02452	Collagen alpha-1(I) chain OS=Homo sapiens GN=COL1A1	D	RGDAGPK	G	0.000	0.000	0.487	0.494
P02461	Collagen alpha-1(III) chain OS=Homo sapiens GN=COL3A1	R	GAPGPAGPRGAAGEPG	R	0.001	0.000	0.516	0.495
P16112	Aggrecan core protein OS=Homo sapiens GN=ACAN	A	PGLPSATPTASGDR	T	159.752	114.257	0.715	0.499
P02452	Collagen alpha-1(I) chain OS=Homo sapiens GN=COL1A1	I	GNVGAPGAK	G	0.003	0.003	0.831	0.501
P20908	Collagen alpha-1(V) chain OS=Homo sapiens GN=COL5A1	S	ILTTVKAK	K	0.002	0.001	0.415	0.501
P02461	Collagen alpha-1(III) chain OS=Homo sapiens GN=COL3A1	K	GAAGPPGPPGAAGTPGLQG	M	1.107	0.941	0.850	0.502
P02461	Collagen alpha-1(III) chain OS=Homo sapiens GN=COL3A1	G	APGPAGSRGAPGPQGPR	G	0.554	0.471	0.850	0.502
P02461	Collagen alpha-1(III) chain OS=Homo sapiens GN=COL3A1	P	GAPGLMGAR	G	2.755	2.269	0.824	0.504
P20908	Collagen alpha-1(V) chain OS=Homo sapiens GN=COL5A1	R	DGLQGPVGLPGPAGPVGPPGEDGDKGEIGE	P	0.725	0.602	0.830	0.504
P20908	Collagen alpha-1(V) chain OS=Homo sapiens GN=COL5A1	G	APGKPGPDGLR	G	0.120	0.104	0.872	0.505
P20908	Collagen alpha-1(V) chain OS=Homo sapiens GN=COL5A1	G	EIGEPGQKGSK	G	0.376	0.313	0.832	0.505
P02461	Collagen alpha-1(III) chain OS=Homo sapiens GN=COL3A1	G	SPGVSGPKGDAGQPGEK	G	0.037	0.029	0.794	0.505
P20908	Collagen alpha-1(V) chain OS=Homo sapiens GN=COL5A1	G	AQGLPGPQGAIGPPGEK	G	297.202	206.322	0.694	0.507
P02461	Collagen alpha-1(III) chain OS=Homo sapiens GN=COL3A1	K	GDAGAPGAPGGKG	D	0.000	0.000	0.443	0.509
P02461	Collagen alpha-1(III) chain OS=Homo sapiens GN=COL3A1	K	GEPGGPGADGVPGKDGP	R	1.624	1.306	0.804	0.509
P02452	Collagen alpha-1(I) chain OS=Homo sapiens GN=COL1A1	L	GAPGPSGAR	G	0.006	0.004	0.772	0.513
P20908	Collagen alpha-1(V) chain OS=Homo sapiens GN=COL5A1	K	GGQGPPGPQGP	I	15.394	12.723	0.827	0.514
P02461	Collagen alpha-1(III) chain OS=Homo sapiens GN=COL3A1	K	GEMGPAGIPGAPGLMGARG	P	0.485	0.426	0.877	0.517
P49747	Cartilage oligomeric matrix protein OS=Homo sapiens GN=COMP	G	VACIQTESGAR	C	0.450	0.387	0.861	0.518
P02461	Collagen alpha-1(III) chain OS=Homo sapiens GN=COL3A1	G	QPGVMGFPGPKGNDGAPGK	N	0.001	0.001	0.865	0.520
P02452	Collagen alpha-1(I) chain OS=Homo sapiens GN=COL1A1	P	GIAGAPGFPGARGPSGPQGPGGPPGPK	G	0.016	0.010	0.587	0.523
P20908	Collagen alpha-1(V) chain OS=Homo sapiens GN=COL5A1	Q	GEPGPPGQQGNPGAQGLPGPQGAIGPPGEK	G	104.732	93.079	0.889	0.528
P02452	Collagen alpha-1(I) chain OS=Homo sapiens GN=COL1A1	R	GEPGPTGLPGPPGERGGP	G	0.019	0.016	0.855	0.534
P20908	Collagen alpha-1(V) chain OS=Homo sapiens GN=COL5A1	G	PSGAPGADGPQGPPGGIGNPGAVGEK	G	0.329	0.259	0.787	0.541

P20908	Collagen alpha-1(V) chain OS=Homo sapiens GN=COL5A1	P	GEKGHR	G	0.039	0.030	0.778	0.542
P02461	Collagen alpha-1(III) chain OS=Homo sapiens GN=COL3A1	N	GQGPQGPK	G	0.296	0.197	0.663	0.543
P02461	Collagen alpha-1(III) chain OS=Homo sapiens GN=COL3A1	R	NGDPGIPG	Q	0.000	0.000	0.167	0.544
P20908	Collagen alpha-1(V) chain OS=Homo sapiens GN=COL5A1	K	GETGDVGQMGPPGPPGPRGPSGAPGADGPQGPPGGIGNPGA	V	1.780	1.159	0.651	0.548
P20908	Collagen alpha-1(V) chain OS=Homo sapiens GN=COL5A1	P	IGPPGPPGLPGPPGPK	G	72.043	53.323	0.740	0.554
P20908	Collagen alpha-1(V) chain OS=Homo sapiens GN=COL5A1	P	AGKPGRR	G	0.002	0.001	0.562	0.556
P49747	Cartilage oligomeric matrix protein OS=Homo sapiens GN=COMP	K	VVDKIDVCP	E	0.151	0.133	0.886	0.561
P02461	Collagen alpha-1(III) chain OS=Homo sapiens GN=COL3A1	P	GPKGDAGAPGAPGGK	G	179.112	126.049	0.704	0.563
P02452	Collagen alpha-1(I) chain OS=Homo sapiens GN=COL1A1	R	GETGPAGRPGEVGPPGPPGP	A	0.098	0.068	0.696	0.567
P02461	Collagen alpha-1(III) chain OS=Homo sapiens GN=COL3A1	P	VGPSGPPGK	D	0.000	0.000	0.586	0.568
P02461	Collagen alpha-1(III) chain OS=Homo sapiens GN=COL3A1	P	GQNGEPGGK	G	0.001	0.001	0.752	0.572
P20908	Collagen alpha-1(V) chain OS=Homo sapiens GN=COL5A1	D	PGPLGPPGEKGK	L	0.107	0.069	0.644	0.573
P49747	Cartilage oligomeric matrix protein OS=Homo sapiens GN=COMP	G	PGEQLR	N	0.052	0.041	0.806	0.575
P20908	Collagen alpha-1(V) chain OS=Homo sapiens GN=COL5A1	G	EKGAPGEK	G	0.016	0.014	0.900	0.576
P20908	Collagen alpha-1(V) chain OS=Homo sapiens GN=COL5A1	G	PQGAIGPPGEK	G	0.841	0.718	0.853	0.581
Q06828	Fibromodulin OS=Homo sapiens GN=FMOD	G	SPSPPDPR	D	0.006	0.004	0.592	0.584
P02452	Collagen alpha-1(I) chain OS=Homo sapiens GN=COL1A1	K	AGERGVPGP	P	0.001	0.000	0.715	0.586
P02461	Collagen alpha-1(III) chain OS=Homo sapiens GN=COL3A1	K	GDAGAPGERGP	P	0.037	0.032	0.862	0.587
P20908	Collagen alpha-1(V) chain OS=Homo sapiens GN=COL5A1	P	RGLPGEPGPR	G	0.080	0.050	0.631	0.587
P02452	Collagen alpha-1(I) chain OS=Homo sapiens GN=COL1A1	R	GLPGTAGLPG	M	0.003	0.002	0.823	0.589
P20908	Collagen alpha-1(V) chain OS=Homo sapiens GN=COL5A1	R	SSKGPDV	A	0.004	0.003	0.661	0.591
P20908	Collagen alpha-1(V) chain OS=Homo sapiens GN=COL5A1	P	KGPPGPPGK	D	0.018	0.014	0.758	0.591
P20908	Collagen alpha-1(V) chain OS=Homo sapiens GN=COL5A1	R	GFPGDRGL	P	0.001	0.001	0.657	0.592
P02452	Collagen alpha-1(I) chain OS=Homo sapiens GN=COL1A1	E	KAHDGGR	Y	0.001	0.001	0.779	0.593
P20908	Collagen alpha-1(V) chain OS=Homo sapiens GN=COL5A1	I	GQPGPSGADGEPGPR	G	0.005	0.004	0.863	0.594
P02461	Collagen alpha-1(III) chain OS=Homo sapiens GN=COL3A1	G	PQGVKGER	G	25.507	19.676	0.771	0.596
P02452	Collagen alpha-1(I) chain OS=Homo sapiens GN=COL1A1	P	AGRPGEVGPPGPPGPAGEKGSPGADGPAGAPGTPGPQGIAGQR	G	4.874	3.418	0.701	0.597
P20908	Collagen alpha-1(V) chain OS=Homo sapiens GN=COL5A1	D	GPQGPPGGIGNPGAVGEK	G	6.339	5.722	0.903	0.608
P20908	Collagen alpha-1(V) chain OS=Homo sapiens GN=COL5A1	K	GNEGPPGPPG	P	7.651	6.020	0.787	0.611
P02452	Collagen alpha-1(I) chain OS=Homo sapiens GN=COL1A1	R	GPAGPPGRDGIPGQ	P	0.017	0.015	0.852	0.614
P02461	Collagen alpha-1(III) chain OS=Homo sapiens GN=COL3A1	P	PGGSGPAGPPGPQGVK	G	0.059	0.051	0.871	0.617
P07585	Decorin OS=Homo sapiens GN=DCN	S	LANTPHLR	E	0.299	0.259	0.868	0.617
Q06828	Fibromodulin OS=Homo sapiens GN=FMOD	E	HNNLTR	V	0.662	0.581	0.877	0.621
P20908	Collagen alpha-1(V) chain OS=Homo sapiens GN=COL5A1	Q	DGPPGDKGDDGEPGQTGSPGPTGEPGPSGPPGK	R	0.774	0.627	0.809	0.624
P02461	Collagen alpha-1(III) chain OS=Homo sapiens GN=COL3A1	G	ERGLPGPPGIK	G	12.335	10.177	0.825	0.629
P20908	Collagen alpha-1(V) chain OS=Homo sapiens GN=COL5A1	Y	PGPRGVK	G	0.000	0.000	0.252	0.632
P02461	Collagen alpha-1(III) chain OS=Homo sapiens GN=COL3A1	V	PGFPGMK	G	0.066	0.039	0.595	0.634
P20908	Collagen alpha-1(V) chain OS=Homo sapiens GN=COL5A1	P	TGFPGPKGPPGPPGK	D	62.039	49.875	0.804	0.635
P20908	Collagen alpha-1(V) chain OS=Homo sapiens GN=COL5A1	P	KGPPGPPGK	D	0.016	0.012	0.767	0.643
P02461	Collagen alpha-1(III) chain OS=Homo sapiens GN=COL3A1	S	GPPGKDGTSGHPGPIGPPGPR	G	0.426	0.400	0.939	0.643
P02461	Collagen alpha-1(III) chain OS=Homo sapiens GN=COL3A1	G	DKGETGER	G	0.004	0.002	0.646	0.647
P02452	Collagen alpha-1(I) chain OS=Homo sapiens GN=COL1A1	G	DAGPPGPAGPAGPPGPIGNVGAPGAKGAR	G	0.095	0.089	0.929	0.652
P02452	Collagen alpha-1(I) chain OS=Homo sapiens GN=COL1A1	R	GVQGPPGPAG	P	0.000	0.000	0.651	0.652
P20908	Collagen alpha-1(V) chain OS=Homo sapiens GN=COL5A1	K	GPPGPPGKDG	L	0.000	0.000	0.450	0.654
P02452	Collagen alpha-1(I) chain OS=Homo sapiens GN=COL1A1	R	GRPGAPGPAGARGNDGATGAAGP	P	0.034	0.030	0.873	0.655
P02461	Collagen alpha-1(III) chain OS=Homo sapiens GN=COL3A1	K	GSPGAQGPPGAPGPLGIAG	I	0.313	0.283	0.905	0.657
P20908	Collagen alpha-1(V) chain OS=Homo sapiens GN=COL5A1	E	GVQGP	D	0.001	0.001	0.855	0.659
P02452	Collagen alpha-1(I) chain OS=Homo sapiens GN=COL1A1	R	GAAGLPGPKG	D	0.143	0.128	0.895	0.661
P07585	Decorin OS=Homo sapiens GN=DCN	T	SIPQGLPPSLTELHLDGNK	I	4.655	4.249	0.913	0.665
P02452	Collagen alpha-1(I) chain OS=Homo sapiens GN=COL1A1	K	GANGAPGIAGA	P	0.002	0.001	0.842	0.666
P20908	Collagen alpha-1(V) chain OS=Homo sapiens GN=COL5A1	K	TVLEIDTPKVEQV	P	0.015	0.014	0.911	0.670
P02461	Collagen alpha-1(III) chain OS=Homo sapiens GN=COL3A1	T	SLKSVNGQIESLISPDGSR	K	6.509	4.961	0.762	0.675
P20908	Collagen alpha-1(V) chain OS=Homo sapiens GN=COL5A1	K	GNEGPPGPPGPAGSPGERGPA	G	10.982	9.663	0.880	0.677
P02461	Collagen alpha-1(III) chain OS=Homo sapiens GN=COL3A1	R	GLAGPPGMPGPRGSPGPQGV	K	0.047	0.032	0.672	0.677
P02461	Collagen alpha-1(III) chain OS=Homo sapiens GN=COL3A1	G	DKGEGGAPGLPGIAGPR	G	1781.989	1465.330	0.822	0.684
P02461	Collagen alpha-1(III) chain OS=Homo sapiens GN=COL3A1	P	GEKGSPGAQGPPGAPGPLGIAGITGAR	G	254.720	228.126	0.896	0.685
P02461	Collagen alpha-1(III) chain OS=Homo sapiens GN=COL3A1	G	PAGIPGFPGMKGHR	G	3.273	2.865	0.875	0.688
P20908	Collagen alpha-1(V) chain OS=Homo sapiens GN=COL5A1	P	PGEPGPR	K	0.001	0.000	0.753	0.696
P02461	Collagen alpha-1(III) chain OS=Homo sapiens GN=COL3A1	G	FPGSPGAK	G	0.000	0.000	0.834	0.697
P20908	Collagen alpha-1(V) chain OS=Homo sapiens GN=COL5A1	P	KGPPGPPGK	D	0.042	0.036	0.869	0.698
P02452	Collagen alpha-1(I) chain OS=Homo sapiens GN=COL1A1	P	GLPGPPGA	K	0.004	0.003	0.800	0.703
P02461	Collagen alpha-1(III) chain OS=Homo sapiens GN=COL3A1	P	GMPGPRGSPGPQGVK	G	1.115	0.996	0.893	0.705
P02461	Collagen alpha-1(III) chain OS=Homo sapiens GN=COL3A1	R	GPVGPSGPP	G	0.012	0.011	0.920	0.705
P02452	Collagen alpha-1(I) chain OS=Homo sapiens GN=COL1A1	K	GDRGETGPAGPPGAPGAP	G	0.072	0.060	0.833	0.713
P02452	Collagen alpha-1(I) chain OS=Homo sapiens GN=COL1A1	R	GVQGPPGPA	G	0.000	0.000	0.700	0.714
P02452	Collagen alpha-1(I) chain OS=Homo sapiens GN=COL1A1	K	GANGAPGIAG	A	0.001	0.001	0.853	0.714
P20908	Collagen alpha-1(V) chain OS=Homo sapiens GN=COL5A1	P	IGIPGRPGPQGPPGPAGEKGAPGEK	G	25550.517	20646.296	0.808	0.715
Q06828	Fibromodulin OS=Homo sapiens GN=FMOD	L	YLQGNR	I	0.076	0.062	0.819	0.717
P02461	Collagen alpha-1(III) chain OS=Homo sapiens GN=COL3A1	G	DAGAPGAPGGKGDAGAPGER	G	0.007	0.006	0.789	0.720
P16112	Aggrecan core protein OS=Homo sapiens GN=ACAN	K	VSTAGDISGATPVLPGSGVEV	S	0.004	0.003	0.882	0.721
P02452	Collagen alpha-1(I) chain OS=Homo sapiens GN=COL1A1	G	EVGPPGPPGPAGEK	G	0.125	0.116	0.925	0.728
P02452	Collagen alpha-1(I) chain OS=Homo sapiens GN=COL1A1	R	GEPGPPGPAGFAGPPGADG	Q	1.226	1.035	0.844	0.728
P02452	Collagen alpha-1(I) chain OS=Homo sapiens GN=COL1A1	R	GRPGAPGPAGARGND	G	0.012	0.011	0.904	0.731
P20908	Collagen alpha-1(V) chain OS=Homo sapiens GN=COL5A1	A	GHPGPPGPPGPPGEVIQPLPIQASR	T	193.582	159.109	0.822	0.733
P02452	Collagen alpha-1(I) chain OS=Homo sapiens GN=COL1A1	G	PGGPPGPKGNSGEPGAPGSK	G	0.065	0.058	0.887	0.735
P02461	Collagen alpha-1(III) chain OS=Homo sapiens GN=COL3A1	K	GHRGFPGNPGAPGSPGPAGQQGAIG	S	0.469	0.436	0.930	0.738
P20908	Collagen alpha-1(V) chain OS=Homo sapiens GN=COL5A1	P	PGPAGKPGR	R	0.000	0.000	0.728	0.739
P02452	Collagen alpha-1(I) chain OS=Homo sapiens GN=COL1A1	A	RGAPGDR	G	0.003	0.003	0.883	0.742
P02461	Collagen alpha-1(III) chain OS=Homo sapiens GN=COL3A1	R	DGVPGGPGMRGMPGSPGGPGSDGKP	G	3.158	2.896	0.917	0.744
P02461	Collagen alpha-1(III) chain OS=Homo sapiens GN=COL3A1	E	PGGPGADGVPGK	D	0.695	0.648	0.933	0.749
P02452	Collagen alpha-1(I) chain OS=Homo sapiens GN=COL1A1	Q	GPPGPPGSPGEQGPSGASGPAGPR	G	0.016	0.013	0.808	0.755
P02452	Collagen alpha-1(I) chain OS=Homo sapiens GN=COL1A1	P	GAPGAPGAPGPVGPAGK	S	0.000	0.000	0.821	0.758
P20908	Collagen alpha-1(V) chain OS=Homo sapiens GN=COL5A1	R	GPSGAPGADGPQGPPGGIGNPGAV	G	0.155	0.123	0.798	0.759
P02452	Collagen alpha-1(I) chain OS=Homo sapiens GN=COL1A1	G	SPGPAGPK	G	0.019	0.017	0.912	0.759
P20908	Collagen alpha-1(V) chain OS=Homo sapiens GN=COL5A1	P	PGKDGLPGHPGQR	G	0.031	0.028	0.920	0.761
P49747	Cartilage oligomeric matrix protein OS=Homo sapiens GN=COMP	A	ALQDVR	E	0.007	0.007	0.925	0.761
P20908	Collagen alpha-1(V) chain OS=Homo sapiens GN=COL5A1	R	QGPKGSIGFPGFP	G	0.042	0.037	0.892	0.765
P02461	Collagen alpha-1(III) chain OS=Homo sapiens GN=COL3A1	G	APGGKGDAGAPGER	G	17.545	14.149	0.806	0.770
P49747	Cartilage oligomeric matrix protein OS=Homo sapiens GN=COMP	W	QANPFR	A	0.012	0.010	0.823	0.785
P02452	Collagen alpha-1(I) chain OS=Homo sapiens GN=COL1A1	V	QGPPGPAGEEGK	R	0.008	0.007	0.896	0.804
P20908	Collagen alpha-1(V) chain OS=Homo sapiens GN=COL5A1	A	GHPGPPGPPGPPGEVIQPLPIQASRTR	R	9.394	8.247	0.878	0.805
P02461	Collagen alpha-1(III) chain OS=Homo sapiens GN=COL3A1	E	AGIPGVPGAK	G	0.013	0.013	0.960	0.806
P02452	Collagen alpha-1(I) chain OS=Homo sapiens GN=COL1A1	G	SKGDTGAK	G	0.115	0.105	0.920	0.807
P20908	Collagen alpha-1(V) chain OS=Homo sapiens GN=COL5A1	P	PGPPGKDGLPGHPGQR	G	0.029	0.027	0.949	0.807
P02461	Collagen alpha-1(III) chain OS=Homo sapiens GN=COL3A1	R	GGAGPPGPEGGKGAAGPPGPPGAAGTPGLQGMPG	E	0.088	0.083	0.942	0.809
P02461	Collagen alpha-1(III) chain OS=Homo sapiens GN=COL3A1	P	GPAGNTGAPGSPGVSGPK	G	0.000	0.000	0.921	0.811
P20908	Collagen alpha-1(V) chain OS=Homo sapiens GN=COL5A1	R	GLPGPVGALGLKGNEGPPGPPGPAGS	P	44407.660	38980.493	0.878	0.813
P02461	Collagen alpha-1(III) chain OS=Homo sapiens GN=COL3A1	K	GEMGPAGIPGAPGLMGARG	P	2.101	1.969	0.937	0.813
P16112	Aggrecan core protein OS=Homo sapiens GN=ACAN	R	YPIVTPRPA	C	10.164	8.379	0.824	0.814
P20908	Collagen alpha-1(V) chain OS=Homo sapiens GN=COL5A1	A	PGADGPQGPPGGIGNPGAVGEK	G	1.359	1.170	0.861	0.814
P20908	Collagen alpha-1(V) chain OS=Homo sapiens GN=COL5A1	K	GETGDVGQMGPPGPPGPRGPSGAPGADGPQ	G	121.520	96.399	0.793	0.814
P02461	Collagen alpha-1(III) chain OS=Homo sapiens GN=COL3A1	G	PAGIPGFPGMKGHR	G	61.850	56.649	0.916	0.828
P02461	Collagen alpha-1(III) chain OS=Homo sapiens GN=COL3A1	R	GLAGPPGM	P	0.039	0.036	0.915	0.828
P20908	Collagen alpha-1(V) chain OS=Homo sapiens GN=COL5A1	R	GIPGPVGEQGLPGSPGPDGP	P	169.481	150.554	0.888	0.833
P02461	Collagen alpha-1(III) chain OS=Homo sapiens GN=COL3A1	K	FCHPELKSGEYWVDPN	Q	6.859	6.312	0.920	0.836
P02461	Collagen alpha-1(III) chain OS=Homo sapiens GN=COL3A1	E	RGAPGFR	G	0.005	0.005	0.910	0.837
P02461	Collagen alpha-1(III) chain OS=Homo sapiens GN=COL3A1	K	NGETGPQGPPGPTGP	G	0.002	0.002	0.948	0.838

P20908	Collagen alpha-1(V) chain OS=Homo sapiens GN=COL5A1	K	GNEGPPGPPGPAGSPGERGPAGAAGPIGIPGRPG	P	3.220	2.917	0.906	0.849
P02461	Collagen alpha-1(III) chain OS=Homo sapiens GN=COL3A1	G	EPGANGLPGAAGER	G	0.000	0.000	0.905	0.852
P02461	Collagen alpha-1(III) chain OS=Homo sapiens GN=COL3A1	R	GPAGPNGIPGEKG	P	0.021	0.019	0.949	0.856
P02461	Collagen alpha-1(III) chain OS=Homo sapiens GN=COL3A1	G	PPGPRGNR	G	0.090	0.079	0.873	0.857
P02461	Collagen alpha-1(III) chain OS=Homo sapiens GN=COL3A1	G	PGADGVPGKDGPR	G	79.918	72.954	0.913	0.859
P02461	Collagen alpha-1(III) chain OS=Homo sapiens GN=COL3A1	R	GSPGGPGAAGFPGARGLPGPPGSNGNPGPPGPSGSPG	K	0.321	0.307	0.956	0.865
P02461	Collagen alpha-1(III) chain OS=Homo sapiens GN=COL3A1	K	FCHPELKSGEYWVDPN	Q	0.057	0.055	0.960	0.866
P16112	Aggrecan core protein OS=Homo sapiens GN=ACAN	P	QPSPLR	V	0.001	0.001	0.813	0.867
P20908	Collagen alpha-1(V) chain OS=Homo sapiens GN=COL5A1	E	GIGGPRGEK	G	0.000	0.000	0.843	0.867
P16112	Aggrecan core protein OS=Homo sapiens GN=ACAN	R	GISAVPSP	G	0.005	0.005	0.914	0.869
P02461	Collagen alpha-1(III) chain OS=Homo sapiens GN=COL3A1	T	SGHPGPIGPPGPR	G	0.042	0.040	0.946	0.870
P02452	Collagen alpha-1(I) chain OS=Homo sapiens GN=COL1A1	R	GPPGSAGAPGKD	G	0.039	0.038	0.975	0.871
P02452	Collagen alpha-1(I) chain OS=Homo sapiens GN=COL1A1	K	GEPGPPGPAG	E	0.001	0.001	0.852	0.872
P20908	Collagen alpha-1(V) chain OS=Homo sapiens GN=COL5A1	A	GEPGLPGEGGPPGPK	G	0.003	0.003	0.945	0.872
P02461	Collagen alpha-1(III) chain OS=Homo sapiens GN=COL3A1	P	SGPRGQPGVMGFPGPK	G	5.924	5.688	0.960	0.878
P02461	Collagen alpha-1(III) chain OS=Homo sapiens GN=COL3A1	S	PGPMGPR	G	0.001	0.001	0.891	0.880
P20908	Collagen alpha-1(V) chain OS=Homo sapiens GN=COL5A1	L	SGPKGEK	G	0.001	0.001	0.956	0.894
P02452	Collagen alpha-1(I) chain OS=Homo sapiens GN=COL1A1	A	GPPGAPGAPGAPGPVGPAGKSGDR	G	0.005	0.005	0.957	0.901
P02461	Collagen alpha-1(III) chain OS=Homo sapiens GN=COL3A1	D	GVPGKDGPR	G	0.001	0.001	0.906	0.902
P02461	Collagen alpha-1(III) chain OS=Homo sapiens GN=COL3A1	G	AGPPGPEGGK	G	0.004	0.004	0.976	0.903
P02461	Collagen alpha-1(III) chain OS=Homo sapiens GN=COL3A1	K	GDTGPPGPQGLQGLPGTGGPPGENGKPG	E	0.025	0.024	0.935	0.905
P16112	Aggrecan core protein OS=Homo sapiens GN=ACAN	L	PSATPTASGDR	T	6.327	5.888	0.931	0.906
P20908	Collagen alpha-1(V) chain OS=Homo sapiens GN=COL5A1	G	TPGKPGPR	G	0.000	0.000	0.947	0.912
P02452	Collagen alpha-1(I) chain OS=Homo sapiens GN=COL1A1	P	PGPAGAAGPAGNPGADGQPGAK	G	0.158	0.152	0.962	0.912
P02452	Collagen alpha-1(I) chain OS=Homo sapiens GN=COL1A1	R	GETGPAGPPGAPGAPGAPGPVGPAGKSG	D	0.072	0.071	0.979	0.926
P02461	Collagen alpha-1(III) chain OS=Homo sapiens GN=COL3A1	P	PGAPGPLGIAGITGARGLAGPPGMPGPR	G	5.918	5.769	0.975	0.928
P16112	Aggrecan core protein OS=Homo sapiens GN=ACAN	V	GDKDSSPGVR	T	0.149	0.142	0.956	0.929
P16112	Aggrecan core protein OS=Homo sapiens GN=ACAN	K	VSTAGDISGATPVLPGSGVEVSSVPESSSETSAY	P	0.584	0.574	0.984	0.937
P02452	Collagen alpha-1(I) chain OS=Homo sapiens GN=COL1A1	K	DGEAGAQGPPGPAGPAGERGEQG	P	0.008	0.008	0.986	0.941
P20908	Collagen alpha-1(V) chain OS=Homo sapiens GN=COL5A1	L	QGPVGLPGPAGPVGPPGEDGDK	G	3.713	3.666	0.987	0.944
P20908	Collagen alpha-1(V) chain OS=Homo sapiens GN=COL5A1	A	IGPPGPR	G	0.159	0.157	0.983	0.946
P20908	Collagen alpha-1(V) chain OS=Homo sapiens GN=COL5A1	P	PGDDGPK	G	0.001	0.001	0.936	0.949
P02452	Collagen alpha-1(I) chain OS=Homo sapiens GN=COL1A1	S	GPMGPRGPPGPPGK	N	0.006	0.006	0.968	0.951
P02461	Collagen alpha-1(III) chain OS=Homo sapiens GN=COL3A1	A	IGPSGPAGK	D	0.157	0.155	0.985	0.952
P02461	Collagen alpha-1(III) chain OS=Homo sapiens GN=COL3A1	R	GSPGGPGAAGFPGARGLPGPP	G	0.077	0.076	0.986	0.957
P20908	Collagen alpha-1(V) chain OS=Homo sapiens GN=COL5A1	K	GQKGEPAIIEPGML	I	3533.836	3438.668	0.973	0.968
P16112	Aggrecan core protein OS=Homo sapiens GN=ACAN	G	TPSSFPTVSLVDR	T	0.002	0.002	0.991	0.971
Q06828	Fibromodulin OS=Homo sapiens GN=FMOD	Y	SLRELHL	S	0.007	0.007	0.985	0.975
P02452	Collagen alpha-1(I) chain OS=Homo sapiens GN=COL1A1	R	GETGPAGPPGAPGAPGAP	G	0.465	0.462	0.992	0.979
P02461	Collagen alpha-1(III) chain OS=Homo sapiens GN=COL3A1	P	PGMPGPR	G	0.001	0.001	0.985	0.984
P02461	Collagen alpha-1(III) chain OS=Homo sapiens GN=COL3A1	G	PIGPPGPR	G	0.000	0.000	0.988	0.985
P20908	Collagen alpha-1(V) chain OS=Homo sapiens GN=COL5A1	P	GPSGPPGKR	G	0.002	0.002	0.988	0.986
P02452	Collagen alpha-1(I) chain OS=Homo sapiens GN=COL1A1	K	VLCDDVICDETKNCPGAEVPEGECCPVCPDGSE	S	0.151	0.150	0.998	0.994
P02452	Collagen alpha-1(I) chain OS=Homo sapiens GN=COL1A1	R	GVQGPPGPAGPRGANGAPGND	G	0.004	0.004	0.996	0.994
P02452	Collagen alpha-1(I) chain OS=Homo sapiens GN=COL1A1	E	PGPPGPAGAAGPAGNPGADGQPGAK	G	0.000	0.000	0.994	0.995
P02461	Collagen alpha-1(III) chain OS=Homo sapiens GN=COL3A1	R	GPPGPAGANGAPGLRGGAG	E	0.062	0.062	0.999	0.998
P20908	Collagen alpha-1(V) chain OS=Homo sapiens GN=COL5A1	P	KGPPGPPGK	D	0.149	0.149	0.999	0.999
P20908	Collagen alpha-1(V) chain OS=Homo sapiens GN=COL5A1	R	GFPGPPGPVGLQGLPGPPGEKGET	G	19.209	19.196	0.999	0.999
P02452	Collagen alpha-1(I) chain OS=Homo sapiens GN=COL1A1	R	GFSGLQGPPGPPGSPGE	Q	0.394	0.000	0.000	#DIV/0!
P02452	Collagen alpha-1(I) chain OS=Homo sapiens GN=COL1A1	D	AGAPGAPGSQGAPGLQGMPGER	G	1.925	0.002	0.001	#DIV/0!

Supplementary S3. Neopeptides in synovial fluid. Neopeptides were identified using tandem mass spectrometry for matrix components aggrecan, biglycan, decorin, fibromodulin, cartilage oligomatrix protein and collagens. Neopeptide abundance was measured in low and high glucosinolate groups and fold change calculated. Neopeptides with higher abundance in the low glucosinolate group are shown. Neopeptide sequences, corresponding protein and accession identifiers, abundance, fold change and p-values are shown (OS: organism species, GN: gene name), Low glucosinolate *n* =8, high glucosinolate *n*= 9.

Gene symbol	Gene name	Cartilage			Fat		
		Fold Change	p-value	Adjusted p-value	Fold Change	p-value	Adjusted p-value
A2M	Alpha-2-Macroglobulin	1.12	0.523	0.983	0.85	0.276	0.9752
ADAMTS1	ADAM Metallopeptidase With Thrombospondin Type 1 Motif, 1	0.89	0.489	0.983	0.96	0.821	0.9911
ADIPOQ	Adiponectin, C1Q And Collagen Domain Containing	1.40	0.529	0.983	1.02	0.926	0.9911
BMP6	Bone Morphogenetic Protein 6	0.87	0.255	0.983	0.88	0.625	0.9752
BST2	Bone Marrow Stromal Cell Antigen 2	1.15	0.620	0.983	0.89	0.496	0.9752
CCL20	Chemokine (C-C Motif) Ligand 20	1.39	0.540	0.983	0.27	0.114	0.9752
CCL5	Chemokine (C-C Motif) Ligand 5	1.61	0.223	0.983	0.60	0.186	0.9752
CCL7	chemokine (C-C motif) ligand 7 (CCL7), mRNA.	0.36	0.059	0.9505	1.41	0.671	0.9752
CCL8	Chemokine (C-C Motif) Ligand 8	1.71	0.286	0.983	0.80	0.561	0.9752
CD274	CD274 Molecule	1.14	0.646	0.983	1.02	0.936	0.9911
COL1A2	Collagen, Type I, Alpha 2	0.93	0.836	0.983	1.15	0.668	0.9752
COL4A1	Collagen, Type IV, Alpha 1	1.22	0.643	0.983	1.13	0.669	0.9752
COL5A1	Collagen, Type V, Alpha 1	0.90	0.623	0.983	1.43	0.221	0.9752
COL6A1	Collagen, Type VI, Alpha 1	0.94	0.783	0.9981	1.33	0.231	0.9752
COMP	Cartilage Oligomeric Matrix Protein	0.97	0.820	0.9981	1.17	0.668	0.9752
CPXM1	Carboxypeptidase X (M14 Family), Member 1	1.89	0.512	0.983	2.06	0.445	0.9752
CTSD	Cathepsin D	0.93	0.522	0.983	0.82	0.494	0.9752
CXCL10	Chemokine (C-X-C Motif) Ligand 10	1.16	0.802	0.9981	0.33	*0.030*	0.9752
CXCL9	Chemokine (C-X-C Motif) Ligand 9	1.44	0.584	0.983	0.77	0.489	0.9752
EMILIN1	Elastin Microfibril Interfacer 1	0.81	0.210	0.983	1.24	0.473	0.9752
FAIM3	Fas Apoptotic Inhibitory Molecule 3	1.35	0.533	0.983	0.41	0.164	0.9752
FCAMR	Fc Receptor, IgA, IgM, High Affinity	1.41	0.687	0.9831	3.31	0.353	0.9752
FMOD	Fibromodulin	1.01	0.964	0.9981	1.09	0.583	0.9752
FN1	Fibronectin 1	1.16	0.294	0.983	0.75	0.666	0.9752
GBP2	Guanylate Binding Protein 2, Interferon-Inducible	0.87	0.192	0.983	0.99	0.883	0.9911
GBP3	Guanylate Binding Protein 3	1.05	0.806	0.9981	1.25	0.225	0.9752
GCLM	Glutamate-Cysteine Ligase, Modifier Subunit	0.77	0.128	0.983	1.02	0.928	0.9911
GDF11	Growth Differentiation Factor 11	0.94	0.814	0.9981	1.11	0.555	0.9752
GPX3	Glutathione Peroxidase 3	0.87	0.318	0.9505	1.11	0.419	0.9752
HABP2	Hyaluronan Binding Protein 2	0.69	0.251	0.9505	8.55	0.218	0.9752

HAPLN1	Hyaluronan And Proteoglycan Link Protein 1	0.99	0.937	0.9981	0.93	0.917	0.9911
HERC5	HECT And RLD Domain Containing E3 Ubiquitin Protein Ligase 5	0.67	*0.009*	0.9505	0.91	0.647	0.9752
HMOX1	Haem Oxygenase 1	1.34	0.339	0.983	1.15	0.658	0.9752
HSPG2	Heparan Sulfate Proteoglycan 2	1.08	0.526	0.983	1.06	0.710	0.9881
HTRA1	HtrA Serine Peptidase 1	0.93	0.599	0.983	0.97	0.968	0.9911
ICAM1	Intercellular Adhesion Molecule 1	0.99	0.967	0.9981	0.90	0.566	0.9752
IFI27	Interferon, Alpha-Inducible Protein 27	0.96	0.901	0.983	0.84	0.401	0.9752
IFI44	Interferon-Induced Protein 44	1.01	0.977	0.9981	0.83	0.317	0.9752
IFI44L	Interferon-Induced Protein 44-Like	0.91	0.758	0.9831	0.74	0.139	0.9752
IFI6	Interferon, Alpha-Inducible Protein 6	0.81	0.244	0.983	0.89	0.632	0.9752
IFI6	Interferon, Alpha-Inducible Protein 6	0.90	0.564	0.983	0.85	0.474	0.9752
IFIT2	Interferon-Induced Protein With Tetratricopeptide Repeats 2	1.20	0.421	0.983	0.84	0.108	0.9752
IGFBP4	Insulin-Like Growth Factor Binding Protein 4	1.61	*0.029*	0.9287	1.41	0.385	0.9752
IL15RA	Interleukin 15 Receptor, Alpha	0.98	0.909	0.9981	1.25	0.210	0.9752
IL1A	Interleukin 1, Alpha	1.67	0.117	0.983	0.85	0.515	0.9752
IL1B	Interleukin 1, Beta	3.48	*0.014*	0.6507	0.85	0.451	0.9752
IL36G	Interleukin 36, Gamma	0.63	0.421	0.983	1.06	0.940	0.9911
IL6	Interleukin 6	1.67	0.400	0.983	0.88	0.570	0.9752
IL8	Interleukin 8	0.97	0.948	0.9981	0.51	0.101	0.9752
IRF1	Interferon Regulatory Factor 1	0.88	0.499	0.983	0.93	0.555	0.9752
IRX3	Iroquois Homeobox 3	1.43	0.303	0.983	1.51	0.055	0.9752
ITGBL1	Integrin, Beta-Like 1 (With EGF-Like Repeat Domains)	0.97	0.893	0.9981	0.70	0.567	0.9752
ITIH3	Inter-Alpha-Trypsin Inhibitor Heavy Chain 3	0.76	0.613	0.983	0.58	0.253	0.9752
LCN2	Lipocalin 2	2.87	0.335	0.983	0.45	0.317	0.9752
LRP8	Low Density Lipoprotein Receptor-Related Protein 8, Apolipoprotein E Receptor	0.95	0.635	0.9831	0.99	0.981	0.9911
LUM	Lumican	0.78	*0.049*	0.983	1.00	0.991	0.9911
MARCKS	Myristoylated Alanine-Rich Protein Kinase C Substrate	1.23	0.531	0.983	1.01	0.953	0.9911
ME1	Malic Enzyme 1, NADP(+)-Dependent, Cytosolic	0.92	0.390	0.983	1.24	0.315	0.9752
MMP12	Matrix metallopeptidase 12	0.83	0.657	0.983	0.94	0.922	0.9911
MMP13	Matrix metallopeptidase 13	0.86	0.741	0.983	0.40	0.267	0.9752
MMP2	Matrix Metallopeptidase 2	0.97	0.950	0.9981	1.07	0.740	0.9911
MMP3	Matrix Metallopeptidase 3	0.99	0.969	0.9981	0.17	0.295	0.9752

NQO1	NAD(P)H Dehydrogenase, Quinone 1	1.00	0.985	0.9981	1.10	0.764	0.9911
OASL	2'-5'-Oligoadenylate Synthetase-Like	1.50	0.349	0.983	0.74	0.249	0.9752
OGN	Osteoglycin	0.84	0.185	0.983	1.03	0.951	0.9911
PARP14	Poly (ADP-Ribose) Polymerase Family, Member 14	1.00	0.990	0.9981	0.93	0.574	0.9752
PENK	Proenkephalin	0.94	0.908	0.9981	1.37	0.476	0.9752
PIM2	Pim-2 Proto-Oncogene, Serine/Threonine Kinase	1.05	0.707	0.9831	0.99	0.954	0.9911
PIR	Pirin (Iron-Binding Nuclear Protein)	0.97	0.841	0.9981	0.94	0.727	0.9911
POPDC3	Popeye Domain Containing 3	0.82	0.146	0.983	2.66	0.177	0.9752
PRG4	Proteoglycan 4	0.88	0.547	0.983	1.11	0.862	0.9911
PTX3	Pentraxin 3, Long	1.21	0.613	0.983	1.17	0.911	0.9911
RPL13A	Ribosomal Protein L13a	0.91	0.679	0.983	1.16	0.492	0.9752
S100A4	S100 Calcium Binding Protein A4	1.00	0.998	0.9981	1.16	0.508	0.9752
SAA4	Serum Amyloid A4, Constitutive	3.55	0.403	0.983	0.36	0.217	0.9752
SELE	Selectin E	1.04	0.964	0.9981	0.37	0.110	0.9752
SERPIND1	Serpin Peptidase Inhibitor, Clade D (Heparin Cofactor), Member 1	0.78	0.204	0.983	1.13	0.855	0.9911
SFRP1	Secreted Frizzled-Related Protein 1	2.64	0.061	0.9505	0.92	0.685	0.9811
SLC15A3	Solute Carrier Family 15 (Oligopeptide Transporter), Member 3	1.08	0.666	0.983	0.87	0.499	0.9752
SLC40A1	Solute Carrier Family 40 (Iron-Regulated Transporter), Member 1	0.89	0.325	0.983	1.04	0.835	0.9911
SOD3	Superoxide Dismutase 3, Extracellular	1.10	0.453	0.983	0.97	0.874	0.9911
TGFBI	Transforming Growth Factor, Beta-Induced, 68kDa	0.80	0.424	0.9505	1.00	0.991	0.9911
THBS3	Thrombospondin 3	0.87	0.318	0.983	1.15	0.589	0.9752
THBS4	Thrombospondin 4	0.91	0.568	0.9981	0.96	0.925	0.9911
TNFAIP6	Tumor Necrosis Factor, Alpha-Induced Protein 6	0.67	0.099	0.6507	0.71	0.551	0.9752
TNFRSF4	Tumor Necrosis Factor Receptor Superfamily, Member 4	0.90	0.450	0.983	1.13	0.594	0.9752
TNFSF10	Tumor Necrosis Factor (Ligand) Superfamily, Member 10	0.95	0.701	0.9981	0.94	0.701	0.9881
TNFSF11	Tumor Necrosis Factor (Ligand) Superfamily, Member 11	0.85	0.429	0.983	0.52	0.416	0.9752
TRAF1	TNF Receptor-Associated Factor 1	1.09	0.597	0.983	0.91	0.507	0.9752
TSG-6	TNF-Stimulated Gene 6 Protein (TNFAIP6)	0.66	0.094	0.9505	0.68	0.511	0.9752
TXNRD1	Thioredoxin Reductase 1	0.86	0.099	0.9981	1.11	0.316	0.9752
UBD	Ubiquitin D	1.57	0.498	0.983	0.85	0.577	0.9752
UBE2L6	Ubiquitin-Conjugating Enzyme E2L 6	1.04	0.828	0.9981	1.01	0.954	0.9911

Supplementary S4. Gene expression in cartilage and fat tissues. Gene expression was measured using a custom Taqman Low Density Array (TLDA), analysed using DataAsisst Software v3.01 and normalised to ribosomal 18S. Data were adjusted using a false discovery rate of 5%. Gene names and symbols are given with fold change (calculated as high/low glucosinolate) and p-values. Cartilage tissue: Low glucosinolate, n=20, high gluosinolate n=17. Fat tissue: Low glucosinolate, n=13, high gluosinolate n=10.

	Diet		
Nutrient	Low glucosinolate (mean ± 95% CI)	High glucosinolate (mean ± 95% CI)	p-value
Energy kcal (kcal/d)	1805.35 (1600 to 2011)	2128.00 (1856 to 2400)	0.20
Energy kJ (kJ/d)	7595.10 (6732 to 8458)	8940.76 (7806 to 10076)	0.20
Protein (g/d)	74.52 (66.48 to 82.56)	86.85 (74.75 to 98.95)	0.23
Carbohydrate (g/d)	215.85 (187 to 244)	258.71 (225 to 292)	0.20
Sugars (g/d)	97.11 (81.74 to 112.50)	117.56 (93.09 to 142)	0.30
Starch (g/d)	110.48 (94.37 to 126.60)	133.49 (113.50 to 153.5)	0.23
Total Fat (g/d)	69.57 (59.95 to 79.19)	79.19 (63.83 to 94.55)	0.39
Saturates (g/d)	25.10 (21 to 29.19)	30.51 (23.34 to 37.68)	0.30
Monounsaturates (g/d)	23.95 (20.54 to 27.35)	25.79 (21.01 to 30.58)	0.64
Polyunsaturates (g/d)	11.14 (9.42 to 12.86)	11.31 (9.20 to 13.41)	0.94
Fibre AOAC (g)	19.19 (17.28 to 21.09)	23.02 (20.02 to 26.01)	0.20
NMES (g/d)	49.79 (37.58 to 62.01)	63.06 (36.24 to 89.88)	0.48
Alcohol (g/d)	8.85 (2.56 to 15.13)	12.76 (2.56 to 22.96)	0.64
Sodium (mg/d)	2720.05 (1982 to 3458)	6486.65 (-548.6 to 13522)	0.37
Potassium (mg/d)	3429.95 (2986 to 3874)	4488.41 (3304 to 5673)	0.23
Calcium (mg/d)	908.10 (774.5 to 1042)	1063.47 (862.9 to 1264)	0.30
Magnesium (mg)	301.00 (263.5 to 338.50)	406.76 (297.8 to 515.7)	0.20
Phosphorus (mg/d)	1324.25 (1185 to 1464)	1598.47 (1356 to 1841)	0.20
Iron (mg/d)	11.89 (10.29 to 13.49)	14.00 (1.27 to 16.72)	0.30
Copper (mg/d)	1.41 (1.07 to 1.75)	2.17 (0.99 to 3.35)	0.30
Zinc (mg/d)	8.56 (7.54 to 9.60)	10.96 (8.64 to 13.27)	0.20
Chloride (mg/d)	3732.95 (3094 to 4372)	4998.76 (3153 to 6844)	0.30
Manganese (mg/d)	3.84 (3.18 to 4.51)	5.30 (4.21 to 6.40)	0.20
Selenium (µg/d)	39.85 (34.7 to 45)	42.18 (31.83 to 52.52)	0.78
Iodine (µg/d)	152.05 (129 to 175)	195.82 (142.8 to 248.90)	0.25
Retinol (µg/d)	510.50 (223.7 to 797.30)	1154.06 (773.1 to 1535)	0.13
Carotene (µg/d)	3493.15 (2827 to 4159)	3306.18 (2003 to 4609)	0.87
Vitamin D (µg/d)	3.04 (2.32 to 3.80)	3.02 (2.25 to 3.80)	0.98
Vitamin E (mg/d)	6.86 (5.44 to 8.27)	7.66 (6.42 to 8.90)	0.55
Thiamin (mg/d)	1.64 (1.45 to 1.84)	1.73 (1.51 to 1.96)	0.64
Riboflavin (mg/d)	2.01 (1.68 to 2.34)	2.06 (1.70 to 2.43)	0.88
Niacin (mg/d)	19.95 (17.03 to 22.88)	20.63 (17.00 to 24.27)	0.87
Potential Niacin (mg/d)	15.11 (13.43 to 16.80)	17.13 (14.52 to 19.73)	0.30
Vitamin B6 (mg/d)	1.91 (1.66 to 2.15)	2.06 ± 0.66	0.61
Vitamin B12 (mg/d)	5.54 (4.50 to 6.58)	6.76 (4.86 to 8.66)	0.37
Folate (µg/d)	274.90 (234.7 to 315.1)	328.65 (273.8 to 383.5)	0.25

Pantothenate (mg/d)	5.87 (5.00 to 6.74)	5.91 (4.96 to 6.87)	0.97
Biotin (µg/d)	41.27 (32.90 to 49.64)	59.42 (38.02, 80.82)	0.24
Vitamin C (mg/d)	92.85 (74.67 to 111)	196.88 (166.2, 227.6)	*9.96E-06*
Nutrient	Low glucosinolate (mean ± 95% CI)	High glucosinolate (mean ± 95% CI)	p-value

Supplementary S5. Self-reported nutritional intakes. Patients recorded their nutritional intakes for 7 days during intervention. Mean intakes ± 95% confidence intervals are shown with adjusted p-values for 1% false discovery rate. Low glucosinolate n=20, high glucosinolate n=17. Significance level was p<0.05.

Copy number	Low glucosinolate<br>n, (%)	High glucosinolate<br>n, (%)	Total<br>n, (%)
GSTT1			
0	2 (5.4)	1 (2.7)	3 (8.1)
1	0 (0)	0 (0)	0 (0)
2	14 (35.1)	13 (37.8)	27 (73)
3	0 (0)	0 (0)	0 (0)
4	4 (10.8)	3 (8.1)	7 (18.9)
GSTM1			
0	7 (18.9)	10 (27.0)	17 (45.9)
1	0 (0)	0 (0)	0 (0)
2	11 (29.7)	7 (18.9)	18 (48.6)
3	0 (0)	0 (0)	0 (0)
4	2 (5.4)	0 (0)	2 (5.4)
GSTP1			
0	0 (0)	0 (0)	0 (0)
1	0 (0)	0 (0)	0 (0)
2	10 (27.0)	8 (21.6)	18 (48.6)
3	3 (8.1)	5 (13.5)	7 (21.6)
4	6 (16.2)	4 (10.8)	10 (27)
5	1 (2.7)	0 (0)	1 (2.7)

Supplementary S6. Predicted GST copy numbers. Genomic DNA was extracted from plasma and the copy numbers were predicted for glutathione S-transferase variants P1, M1 and T1 (GSTP1, GSTM1, GSTT1). Taqman® Copy Number Assays (Life Technologies) were used and the copy numbers were predicted using Copy Caller® software (Life Technologies). Low glucosinolate n=20, high glucosinolate n=17.
